# Transcriptomic landscapes of tissue-specific color transition in eggplant reveal regulatory roles of lncRNAs and alternative splicing in anthocyanin biosynthesis

**DOI:** 10.3389/fpls.2026.1832029

**Published:** 2026-05-28

**Authors:** Federica De Marchi, Alberto Acquadro, Andrea Moglia, Luciana Gaccione, Giuseppe Mannino, Pietro Gramazio, Lorenzo Barchi, Cinzia Margherita Bertea, Ezio Portis, Jaime Prohens, Cinzia Comino

**Affiliations:** 1 Department of Agricultural, Forest and Food Science, University of Turin, Turin, Italy; 2 Department of Life Sciences and Systems Biology, Innovation Centre, University of Turin, Turin, Italy; 3 Instituto de Conservación y Mejora de la Agrodiversidad Valenciana, Universitat Politècnica de València, Valencia, Spain

**Keywords:** delphinidin-3-rutinoside, MYB, PacBio HiFi, RNA-seq, *Solanum melongena*, Spearman correlation

## Abstract

**Background:**

Color transitions from green to purple in eggplant (*Solanum melongena* L.) flowers and berries result from chlorophyll and anthocyanin accumulation. While structural genes (*CHS*, *CHI*, *F3’H*, *DFR*, *ANS*, *UGT*) and regulatory transcription factors (*MYB*, *bHLH*, *WD40*) in the anthocyanin pathway are well characterized, fine-scale transcriptional regulation is less understood. Long non-coding RNAs (lncRNAs) contribute to pre- and post-transcriptional regulation, whereas alternative splicing (AS) events control exclusively post-transcriptional modifications.

**Methods:**

Here we performed an integrated transcriptomic analysis of mRNAs, lncRNAs and AS events to investigate fine regulation of anthocyanin biosynthesis.

**Results:**

A PacBio assembly of the eggplant cultivar “Black Beauty” genome allowed the computational prediction and expression analysis of all transcripts in fruit peel and flower corolla. We identified 4, 126 lncRNAs and 34, 745 novel transcript isoforms, and tissue-specific regulatory networks revealed multiple lncRNAs and protein-coding genes AS events associated with anthocyanin biosynthesis. Quantification of anthocyanins in both peel and corolla enabled the construction of putative tissue-specific models of anthocyanin transcriptional regulation.

**Discussion:**

This study provides a starting point to improve the knowledge of fine-tuning anthocyanin regulation at the transcriptional level, highlighting tissue-specific lncRNAs and AS events linked to regulatory genes, and offering, to our knowledge, the first evidence of lncRNA involvement in anthocyanin biosynthesis in eggplant.

## 1 Introduction

Anthocyanins are water-soluble secondary metabolites responsible for the pigmentation of different plants’ tissues, including fruits and flowers (Tanaka et al., 2008). Among their functional roles, anthocyanins are essential in protecting plants against abiotic and biotic stressors (An et al., 2020), in the attraction of pollinators (Cappellini et al., 2021) and in modulating the cellular redox balance.

The biosynthetic pathway of anthocyanins (**Supplementary Figure 1**) is a branch of the phenylpropanoid biosynthetic grid and entails a cascade of enzymatic reactions starting from the conversion of phenylalanine into coumaroyl-CoA, catalyzed by the enzymes phenylalanine ammonia-lyase (PAL), cinnamate 4-hydroxylase (C4H) and 4-coumarate-CoA ligase (4CL) (Sunil and Shetty, 2022). The cascade continues toward the flavonoid structural genes, classified into early and late biosynthetic genes (EBGs and LBGs, respectively), leading to stable anthocyanins (Shoeva et al., 2017).

The EBGs lead to the synthesis of dihydroflavonol precursors and other flavonolic compounds, and include *chalcone synthase* (*CHS*), *chalcone isomerase* (*CHI*) and *flavanone 3-hydroxylase* (*F3H*). Specifically, CHS catalyzes the synthesis of naringenin chalcone from 4-coumaroyl-CoA and malonyl-CoA, which is then converted by CHI into naringenin. Subsequently, *F3H* hydroxylates naringenin to form dihydrokaempferol, the central substrate for downstream diversification. The genes responsible for the ultimate modifications in anthocyanin production are included in the LBGs, comprising *flavonoid 3′-hydroxylase* (*F3′H*), *flavonoid 3′, 5′-hydroxylase* (*F3′5′H*), which transform dihydrokaempferol into two other dihydroflavonols, dihydroquercetin or dihydromyricetin, respectively, thus enabling structural diversification of anthocyanins (Manickam et al., 2025). *Dihydroflavonol 4-reductase* (*DFR*) then catalyzes the conversion of these dihydroflavonols into colorless leucoanthocyanidins, which are subsequently transformed by *anthocyanidin synthase* (*ANS*) into the pigmented anthocyanidins. Finally, *flavonoid 3-O-glucosyltransferase* (*UFGT*, also known as GT) stabilizes these molecules by the conjugation of sugar residues, yielding stable anthocyanins, which may undergo additional modifications such as acylation (Lo Scalzo et al., 2021; Manickam et al., 2025).

In dicotyledons, the transcriptional regulation of the anthocyanin pathway is mostly mediated by transcription factors (TFs) forming the MYB-bHLH-WD (MBW) ternary complex, consisting of R2-R3-MYB, basic helix-loop-helix (bHLH) and WD40-repeat proteins (Jaakola, 2013). Besides functioning within the MBW complex, tissue-specific MYB TFs can also positively regulate anthocyanin biosynthesis and accumulation alone. In addition, these TFs could also influence anthocyanin production indirectly through hormone signaling. Particularly, the increase in ethylene hormone and its signaling pathway mediated by *Ethylene Response Factors* (*ERFs*) has been linked to anthocyanin accumulation in several plant species (Yu et al., 2022). In contrast, auxin biosynthesis was found to be antithetical to that of anthocyanins (Li et al., 2024a).

Beyond transcriptional and hormone-mediated regulation, recent advances in high-throughput sequencing technologies and computational analytical approaches have also highlighted the importance of alternative splicing (AS) and long non-coding RNAs (lncRNAs) as key regulators of gene expression. The AS events are generated from the selection of a different splice site by the spliceosome to produce multiple mature mRNAs. AS plays a key role in post-transcriptional regulation, influencing several biological processes in plants, such as both biotic and abiotic stress responses, tissues’ development and morphogenesis (Yan et al., 2021; Mandadi et al., 2023). It has been estimated that up to 60% of intron-containing genes in plants undergo alternative splicing (AS) events, a proportion that has risen to nearly 70% in more recent transcriptomic analyses (Szakonyi and Duque, 2018). These events expand transcript diversity and can therefore alter protein function or create premature termination codons that trigger nonsense-mediated decay, leading to mRNA degradation (Syed et al., 2012; Sibley et al., 2016). In recent studies, some protein-coding genes AS events have been reported as anthocyanin-related among the *Solanaceae* family. In tomato, the alternative 5’ splicing sites of *SlAN2-like* R2-R3 MYB transcription factor lead to the partial loss of the R3 domain that causes a no-anthocyanin phenotype (Colanero et al., 2020). Non-coding RNAs with a length greater than 200 nucleotides are identified as lncRNAs (Heo et al., 2013). These molecules can recruit factors for epigenetic and chromatin modification and can act as pre- and post-transcriptional regulators of target genes, being characterized by a lower expression and conservation between different organisms compared to mRNAs (Wang et al., 2023). The lncRNAs mode of action includes cis- and trans-acting mechanisms, depending on the location of their target genes. LncRNAs regulate several biological processes in plants, including flowering time, crop yield, responses to abiotic and biotic stresses, reproduction, and organogenesis, by interacting with DNA, RNA, and proteins (Li et al., 2023a; Mattick et al., 2023; Li et al., 2024b). In recent years, some works have focused on anthocyanin accumulation and biosynthesis in several plant species. In flowers of *Melastoma candidum*, different lncRNAs have been identified as regulators of *4CL*, *CHS*, *CHI*, *F3H*, and *MYB4* genes (Li et al., 2023b), and in apple fruits, the lncRNA *MdLNC610* has been positively correlated with *MdACO1* transcript, enhancing light-induced anthocyanin accumulation by activating ethylene synthesis (Yu et al., 2022). Similarly, in carrot roots, the lncRNAs *asDcMYB6* and *asDcMYB7* were found to have an expression pattern consistent with the two *MYB* TFs from which they originate, possibly leading to a positive role in the anthocyanin pathway regulation (Chialva et al., 2021).

Eggplant (*Solanum melongena* L.) is the third most cultivated crop in the *Solanaceae* family, after potato and tomato (Martina et al., 2024; FAO, 2025). Unlike tomato and potato, eggplant exhibits several anthocyanin accumulation patterns in its fruit peel and flower petals, ranging from white to purple. Specifically, in eggplant varieties with violet pigmentation, anthocyanin accumulation is restricted to derivatives of delphinidin, which are also detectable in vegetative tissues (Filyushin et al., 2024). In fruit, the abundance of delphinidin is maximal at early developmental stages and progressively declines during fruit maturation, ultimately becoming undetectable at full physiologically ripeness (Panda et al., 2025). The predominant anthocyanins identified are delphinidin-3-(*p*-coumaroyl-rutinoside)-5-glucoside (commonly referred to as nasunin) and delphinidin-3-rutinoside (commonly referred to as tulipanin) (Gaccione et al., 2023). Purple eggplant extracts have been reported to show a high superoxide anion radical scavenging activity, underlying the importance of anthocyanins’ role not only in the regulation of plant physiological processes, but also in human health and nutrition (Noda et al., 2000; Putta et al., 2017; Mattioli et al., 2020). The structural genes and regulatory transcription factors (TFs) of the anthocyanin pathway have been deeply characterized in eggplant, where two major QTLs have been identified on chromosomes 5 and 10 (Barchi et al., 2012; Toppino et al., 2020). In the fruit peel of *S. melongena* accessions, the *MYB1* gene (also known as *MYB113*) has been characterized as the activator of anthocyanin biosynthesis, acting solo or interacting with the bHLH transcription factor *Transparent Testa 8* (*TT8*) in the MBW complex; whereas, in flowers, the MYBs involved in anthocyanin production are reported to be *MYB2* (or *AN2*) and *MYB75* (Shi et al., 2021; Filyushin et al., 2023; Ai et al., 2025). In fruits, *MYBL1*, a R3 type MYB, has also been linked to anthocyanin biosynthesis as a negative regulator in *S. melongena* (Moglia et al., 2020). MYB TFs expression could be regulated by miRNAs, such as miRNA156/157, which are involved in the regulation of anthocyanin biosynthesis in eggplant by directly interacting with MYBs or indirectly by promoting *SQUAMOSA PROMOTER BINDING-LIKE* (*SPL*) genes (Panda et al., 2025).

Despite the extensive characterization of the structural genes and TFs involved in the production of anthocyanins, the fine-tuning at the transcriptomic level, mediated by alternative splicing (AS) events, and especially long non-coding RNAs (lncRNAs), remains largely unexplored in eggplant. An alternative 5’ splicing site of the *DFR* gene in two anthocyanin-free *S. melongena* mutants has been associated with the variation in anthocyanin accumulation observed during the domestication of the spiny *Solanum* group (Wang et al., 2022a).

To our knowledge, no studies have reported the involvement of lncRNAs in the biosynthesis of anthocyanins in eggplant, and protein-coding genes AS has been only partially explored.

Here, we present a comprehensive transcriptomic analysis, focusing on mRNA, lncRNA and AS events to elucidate the fine regulation of the anthocyanin biosynthetic pathway in eggplant fruits and flowers. To this purpose, we performed a PacBio assembly of the “Black Beauty” (BB) cultivar genome, which has purple berries and flowers, and created regulatory networks to explore the role of lncRNA and AS in the biosynthesis and accumulation of anthocyanins, associating all collected data with metabolic analysis.

## 2 Materials and methods

### 2.1 Plant material

Plants of the eggplant cultivar “Black Beauty” (BB) were grown in a greenhouse at a mean temperature of 25 °C for four months. The peel of three berries from three plants was collected and pooled at three developmental stages, yielding a total of nine fruit samples: fruit set surrounded by sepals to ensure no light exposure, displaying green coloration (ST1); enlarged fruits exhibiting color shifting from green to purple (ST2); large commercially mature fruits with fully purple peel (ST3). Similarly, five flower corollas were collected from each sampled plant and were pooled at two stages of development: Stage green (STG), corresponding to close buds with green petals, and Stage purple (STP), corresponding to open buds with purple petals, resulting in a total of six flower samples.

### 2.2 Extraction and analysis of anthocyanin compounds

Anthocyanins were extracted from plant material using an acidified hydroalcoholic solvent. Briefly, samples were homogenized and extracted with 50% (v/v) ethanol acidified with 1% (v/v) formic acid, using a solid-to-solvent ratio of 1:10 (w/v). The extraction was carried out under constant agitation at room temperature, with samples protected from light. Subsequently, the extracts were clarified by centrifugation, and the supernatants were collected for further analysis (Mannino and Maffei, 2022; Mannino et al., 2022). The analysis of anthocyanins was performed using an Agilent Technologies 1200 HPLC system coupled with a DAD and an LC-MS 6330 Series Ion Trap (Agilent Technologies, USA) equipped with an electrospray ionization (ESI) source. Chromatographic separation was performed at a constant flow rate of 0.2 ml min^-1^ on a reverse-phase C18 Luna column (3.00 μm, 150 × 3.0 mm i.d., Phenomenex, USA), maintained at 25 °C using the Agilent 1100 HPLC G1316A column compartment. The solvents employed for the analysis were: (A) water acidified with 0.1% (*v/v*) formic acid and (B) 50% (*v/v*) methanol acidified with 0.1% (*v/v*) formic acid. The linear gradient adopted was: 0–5 min 5% (*v/v*) B, 5–25 min 5–25% (*v/v*) B, 25–35 min 25–40% (*v/v*) B, 35–40 min 40–5% (*v/v*) B, followed by column re-equilibration. UV–VIS spectra were recorded between 220 and 650 nm, while chromatographic profiles were acquired at 220, 280, 360, and 520 nm, the latter specifically for the detection of anthocyanins. Tandem mass spectrometry analyses were performed in positive mode for anthocyanins, using nitrogen as the nebulization gas (flow 5.0 ml min^-1^, temperature 325 °C) and capillary voltage of 1.5 kV; helium was used as the collision gas. The compounds were identified by comparing the retention times and UV–VIS/MS spectra with those of authentic reference compounds or based on data in the literature. The data obtained from HPLC-ESI-DAD-MS/MS analyses were processed and normalized using the MetaboAnalyst platform vs. 24 (www.metaboanalyst.ca). Since no missing values were detected, the Low-quality filter was not applied. However, the low-variance filter based on the interquartile range (IQR) with a threshold of 5% was used to exclude features that were almost constant between experimental conditions. In addition, a low-abundance filter based on the average intensity value of the features was applied. To compensate for systematic differences between samples, the data were normalized by median and transformed using log_2_, a mathematical transformation that also allows negative values to be handled using simple log and square root approaches. Subsequently, each variable was centered on the mean and divided by the square root of the standard deviation (Pareto scaling) to balance the influence of features with different dispersion.

### 2.3 Nucleic acids extraction, quality control, sequencing and qPCR

High-molecular-weight nuclear DNA was extracted from the previously ground and frozen peel of ST2 fruits using a modified CTAB method (Li et al., 2024c). Briefly, 800 µL of extraction buffer (2% (*w/V*) CTAB, 0.1 M Tris-HCl and 20 mM EDTA disodium salt, 1.4 M NaCl) supplemented with PVP40 1% (*w/V*) and 0.2% (*v/v*) β-Mercaptoethanol right before use, were added to 100 mg of frozen tissue powder in a 2 mL Eppendorf tube. The mixture was gently mixed by inverting the tube and 3 µL of RNase A solution (4 mg/mL, Promega) were added. The tube was incubated at 37 °C for 12 minutes. Then, 10 µL of Proteinase K (20 mg/mL, Promega) were added, the tube was inverted a couple of times and incubated at 56 °C for 15 minutes. The sample was kept in ice for 5 minutes, and then 600 µL of cold Phenol-chloroform-isoamyl alcohol (25:24:1) (*v/v/v*) was added. The tube was kept in agitation at room temperature for 10 minutes and centrifugated at 11, 500 rpm for 10 minutes. The supernatant was collected using cut-end tips and placed in a new 2 mL Eppendorf tube, where 42 µL of NH_4_OAC-CH_3_COONH_4_ 5M and, subsequently, 800 µL of pure isopropanol were added. The tube was gently inverted and centrifuged at 13, 000 rpm for 10 minutes at 4 °C to guarantee the DNA pellet formation. The supernatant was discarded and the pellet washed twice in ethanol 70% (*v/v*) followed by a 13, 000 rpm centrifugation for one minute at 4 °C. After the ethanol residue evaporated, 100 µL of ultrapure water was added and the pellet was resuspended at room temperature overnight and then conserved at -20 °C. The total RNA of the six BB corolla and the nine peel samples was extracted using a commercial kit (Spectrum™ Plant Total RNA Kit, Sigma-Aldrich, Milan, Italy), according to the manufacturer’s instructions. DNA and RNA concentration, integrity and quality were assessed by Qubit 2.0 (Life Technologies, Carlsbad, CA, USA), using Qubit dsDNA BR Assay and Qubit dsRNA BR Assay (Life Science), and NanoDrop^®^ ND-1000 UV-Vis Spectrophotometer (Thermo Fisher Scientific) (Martina et al., 2023). To obtain CCS reads (HiFi) for contig assembly, the BB genome was sequenced using a PacBio Revio sequencer (Pacific Biosciences) at Novogene (Beijing, China). The extracted RNA samples were sequenced using Illumina NovaSeq platforms (PE150) at Novogene (Beijing, China). Raw samples’ reads were then cleaned using fastp tool (Chen, 2023).

For the qPCR validation, for the six BB corolla and the nine peel samples, cDNA was synthetized through reverse transcription using 1 µg of extracted RNA and the High-Capacity cDNA Reverse Transcription Kit (Applied Biosystems, USA), following protocol’s instructions. The produced cDNA was diluted 1:6 and used for the following qPCR analyses. qPCR reactions were performed with a final volume of 10 µL using a StepOnePlus Real-Time PCR system (Applied Biosystem), with technical duplicates. Each reaction contained 1, 5 µl of starting cDNA, 2X Power SYBR Green PCR Master Mix (Applied Biosystem, USA) and specific primers (0.2 µM. MYB75 primer For: 5’-CCGCATATCAAGAGAGGTGAC-3’; MYB75 primer Rev: 5’-TGCCTGTTTCCTAGAAGCTTATG-3’. EIL primer For:

5’-TGGGTGTTAGCTGCTAATTCTG-3’; EIL primer Rev: 5’-TCCCCACAGAACCCCATTTC-3’. MSTRG.970.1 primer For: 5’-AGAAGGTGAACGTGGCAGAG-3’; MSTRG.970.1 primer Rev: 5’-ACCTCGATTCCTCAGCTTGG-3’. MSTRG.3938.1 primer For: 5’-TCAATCACCGCCCCTTAACC-3’; MSTRG.3938.1 primer Rev: 5’-TGGGGAAGAAGGGTTTGGTG-3’). The elongation factor (*SmEF*, primer For: 5’-ACCAGCATCACCATTCTTCA-3’; primer Rev: 5’-ACTGCCATACTTCCCACATT-3’) was used as eggplant’s housekeeping gene. The following PCR protocol was used: 1 cycle of 95 °C for 5 min; 40 cycles of 95 °C for 15 s, 60 °C for 60 s. The 2^−ΔΔCt^ method was used for expression quantification based on Ct values of the target genes and the Ct value of the housekeeping gene (*SmEF*). Statistical significance between groups was assessed on ΔCt values using a two-tailed Welch’s t-test, with p < 0.05 considered significant.

### 2.4 Genome assembly and annotation

Hifiasm v0.24 (Cheng et al., 2024) was used to construct a *de novo* assembly of the PacBio HiFi reads and Ragtag scaffold (Alonge et al., 2022) was employed for the assembly scaffolding, using the eggplant genome accession GPE001970 V5 (Gaccione et al., 2025). After using QUAST (Gurevich et al., 2013) to assess the final assembly quality of BB genome, chloroplast and mitochondrial genomes were removed based on their alignment to available eggplant plastidial and mitochondrial genome from NCBI (OP688482.1, OR187866.1), using minimap2 (v2.26). EDTA (Ou et al., 2019) was used to construct a custom repeat library and RepeatMasker was employed to low-mask the BB assembled genome. Subsequently, Helixer v0.3.4 (Holst et al., 2023) and Braker3 v3.0.3n (Gabriel et al., 2024) were used to perform genome annotation. The functional annotation was then conducted on the predicted proteins using the *Viridiplantae* NCBI Reference Sequence Database with diamond blastp algorithm (Buchfink et al., 2021).

BUSCO v6.0 (Manni et al., 2021), with the ‘*solanales_odb10’* dataset, and Merqury v1.3 (Rhie et al., 2020) were used to assess the completeness of the genome assembly.

Minimap2 v2.24 (Li, 2021) was used to align the BB genome against the GPE001970 reference genome and the resulting PAF file was used by pafr R package to produce the dotplot, while GENESPACE v1.4 (Lovell et al., 2022) was used to perform the synteny analysis between the two genomes. CIRCOS (Krzywinski et al., 2009) was used to visualize the topography of the BB genome.

### 2.5 Transcripts alignment and isoform assembly

Filtered reads were aligned with STAR tool (Dobin et al., 2013), using options alignIntronMin 20 and alignIntronMax 20000, and BB assembled genome as a reference. Subsequently, the aligned reads were used as input for StringTie (Shumate et al., 2022) program to obtain the assembled transcripts of peel and corolla replicates. Then, Stringtie merge with default parameters was used to generate the integrated transcript information of all peel and corolla samples. The resulting GTF file was compared with the annotation of BB genome and integrated with the annotation of the assembled transcriptome using GffCompare (Pertea and Pertea, 2020).

### 2.6 Identification of putative lncRNAs and new protein-coding transcripts

To find putative long non-coding RNAs (lncRNAs), the final set of transcripts from GffCompare and BB annotated transcripts were filtered for length > 200 nt and class code “i”, “o”, “s”, “u” and “x”, excluding Chr0. The protein-coding-score was calculated for the resulting set of transcripts using Coding Potential Calculator 2 (CPC2) (Kang et al., 2017), FlExible Extraction of LncRNAs (FEELnc) v0.2.1 (Wucher et al., 2017) and Coding Potential Assessment Tool (CPAT) v3.0.0 (Wang et al., 2013). Moreover, all transcripts with protein-coding domains found in Pfam databases (Paysan-Lafosse et al., 2025) were removed. The transcripts identified as noncoding by all the mentioned tools were considered as the total set of lncRNAs present in BB berries’ peel and flowers’ corolla, while all the transcripts which have passed the filter to be identified as lncRNAs that were conversely classified as coding were listed as novel coding mRNAs.

### 2.7 Detection of protein-coding gene AS events

For the Alternative Splicing (AS) events detection, transcripts with class code “j”, “m” and “n” were selected and used to run SUPPA2 v2.4 (Trincado et al., 2018), excluding Chr0. The local event types generated were the seven main ones: (I) Skipping Exon (SE); (II) Alternative 5’ Splice Sites (A5); (III) Alternative 3’ Splice Sites (A3); (IV) Mutually Exclusive Exons (MX); (V) Retained Intron (RI); (VI) Alternative First Exons (AF); (VII) Alternative Last Exons (AL). The proportion spliced-in (PSI) values were extracted from the ‘psiPerEvent’ of local AS events for each sample and ‘diffSplice’ was performed to calculate the differentially spliced events between two conditions at a time (p-value cutoff: < 0.05).

### 2.8 Phylogenetic analysis of regulatory MYBs and protein domains assessment

BB genome V-myb myeloblastosis viral oncogene homologs (MYBs) predicted protein sequences were obtained using the BLASTP tool to identify known MYBs involved in the regulatory process of anthocyanin biosynthesis as activators or repressors in eggplant (Cammareri et al., 2024). Multiple sequence alignment was performed, including protein sequences of the main anthocyanins activators: *MYB1* (BB_10g017140.1), principal activator in fruits, *AN2* (BB_10g017120.1) and *MYB75* (BB_10g017150.1), principal activators in flowers, *MYB94* (BB_03g025270.1), *MYB19* (BB_09g019020.1), *MYB5* (BB_08g001920.1) and *MYB35* (BB_01g009850.1), other minor activators, detected as positive regulators (**Supplementary File S1**). As negative regulators, *MYBL1* (BB_10g000390.1), the first documented *MYB* repressor, *MYB44* (BB_02g022950.1), and *MYB86* (BB_06g021820.1), minor putative repressors, were included (**Supplementary File S1**). The alignment was performed using MUSCLE algorithm (Edgar, 2004). The visualization of the phylogenetic tree was done by the neighbor-joining algorithm in MEGA11 (Tamura et al., 2021), applying a bootstrap analysis with 1, 000 replicates to assess the statistical significance of nodes.

All *MYBs* TFs protein from BB were also evaluated for the presence of the R2R3 DNA-binding domains using InterPro online tool (https://www.ebi.ac.uk/interpro/).

### 2.9 Differential expression analysis, correlation network construction and evaluation of RNA-RNA interactions

Reads per transcript counts were obtained by re-assembling the lncRNAs, novel coding mRNAs and known coding mRNAs transcripts using StringTie with -e option. The Python script prepDE.py3 (provided by StringTie) was used to generate the raw count matrix. Subsequently, The Differential Expression Analysis (DEA) was conducted using Deseq2 package in R (Love et al., 2014), with a padj threshold set < 0.05 and a value of log2FC set to 1 for coding and novel coding transcripts and to 0.5 for lncRNAs.

The final filtered and normalized count matrix was used to perform the Principal Component Analysis (PCA) for the six corolla and nine peel samples separately.

Heatmaps were generated in R using the pheatmap package (https://CRAN.R-project.org/package=pheatmap) based on variance-stabilized transformed (VST) expression values of significantly differentially expressed transcripts and lncRNAs, and were visualized by hierarchical clustering.

Next, the Transcript Per Million (TPM) normalized matrix was obtained by the function convertCounts from Differential Gene Expression (DGE) Analysis Utility Toolkit (Law et al., 2018) in R. This matrix was employed for the regulatory networks constructions using Spearman correlation coefficient, with a p-value cutoff set < 0.05, to clarify the role of lncRNAs and AS in the biosynthesis and accumulation of anthocyanins. IntaRNA (Mann et al., 2017) was used to evaluate the interaction between strongly correlated alternative spliced transcripts and lncRNAs (correlation cutoff > |0.98| and > |0.9| for corolla and peel, respectively). The temperature parameter was set at 25 °C, reflecting the mean temperature in the greenhouse where the sampled plants were grown, and the results were filtered for a free energy < -30 kcal/mol. RNAfold and RNAcofold Web Servers (Lorenz et al., 2011) were used to visualize the RNAs secondary structures and RNA-RNA interactions. Transcription factors (TFs) known to be involved in anthocyanin regulation that presented an AS event were selected with their respective correlated and interacting lncRNAs (the ten lncRNAs which presented the highest interaction), structural genes of the anthocyanins’ pathway and other known regulatory TFs to obtain the final peel and corolla networks. The correlation values were filtered to keep only interactions with an absolute value greater than 0.8 and 0.7 for corolla and peel, respectively. Cytoscape v3.10.3 (Shannon et al., 2003) was used for networks visualization.

## 3 Results

### 3.1 Black Beauty genome sequencing, assembly and annotation

To achieve the most accurate transcripts identification, especially for long non-coding RNAs (lncRNAs), the complete genome assembly of the cultivar “Black Beauty” (BB) was generated. A total of 84 Gb of PacBio HiFi data were obtained (70X). The assembly generated 1, 399 contigs, with an N50 of 57.2 Mb and an L50 of eight scaffolds, covering a total of 97.13% of the assembled genome. Following RagTag scaffolding using the latest version of the purple accession GPE001970 (“67/3”) as reference, the 12 chromosomes were obtained, for a total genome length of 1.15 Gb, with 20.37 Mb of unplaced sequences (**Supplementary Table 1**).

**Figure 1:**
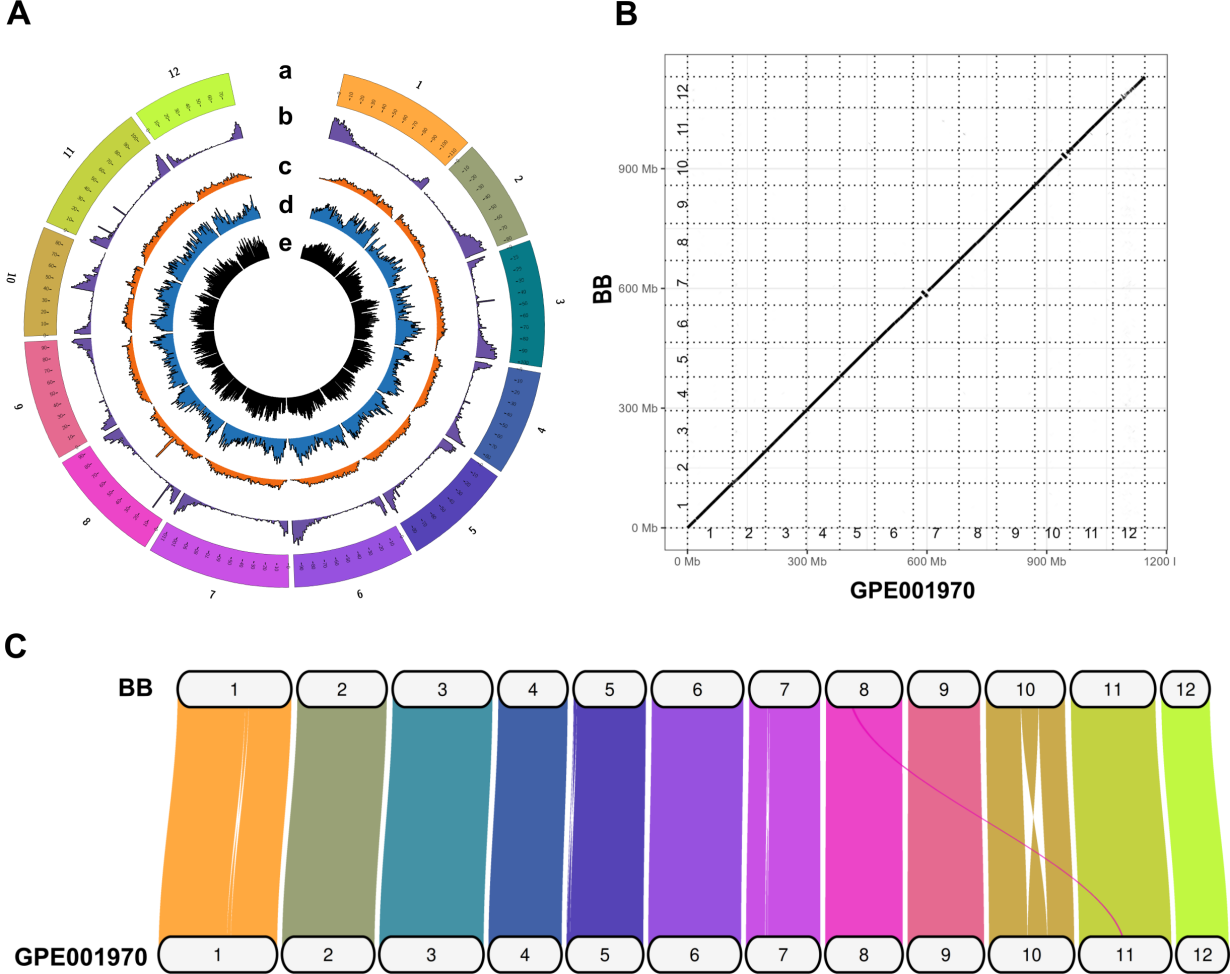
Graphical representation of BB genome and its collinearity with GPE001970. **(A)** Genomic landscape of the eggplant genome. Track **(a)** Eggplant chromosomes; track **(b)** gene density; track **(c)** LTR-Gypsy transposon density; track **(d)** LTR-Copia transposon density; track **(e)** DNA transposon density. For tracks **(b–e)**, densities are shown in 1-Mb intervals. **(B)** Dotplot of the comparisons between BB and GPE001970 genomes. **(C)** Synteny plot of the comparisons between BB and GPE001970 genomes (gene rank).

BUSCO analysis returned 99.3% complete BUSCOs gene copies (97.0% single-copy, 2.3% duplicated), 0.0% fragmented, and 0.7% missing out of 7, 934 genes searched. Merqury k-mer completeness analysis showed that 99.68% of distinct k-mers from the sequencing reads are present in the final assembly, with a consensus quality (QV) of 65, 215 and an error rate of 3.00956e-07 (**Supplementary Table 1**). Following the annotation, 74.76% of the genome was masked, and a total of 31, 316 genes were predicted. To have an overview of BB genomic asset, gene, LTR Gypsy transposon, LTR Copia transposon and DNA transposon density are reported in **Figure 1A**.

We then compared our genome to the reference GPE001970 by synteny analysis, revealing strong collinearity. By investigating whether anthocyanin-related genes conserved their position and order between the two accessions, we found that the only structural variations that could comprehend anthocyanin-related genes was an inversion on chromosome 10 (**Figure 1B**). In particular, the inversion (~7.24 Mb, **Figure 1C**) contained three chalcone isomerase genes (BB_10g014680.1, BB_10g014690.1, and BB_10g014700.1) with no sequence differences relative to the GPE001970 accession.

### 3.2 RNA-seq QC metrics and transcriptome assembly

**Figure 2:**
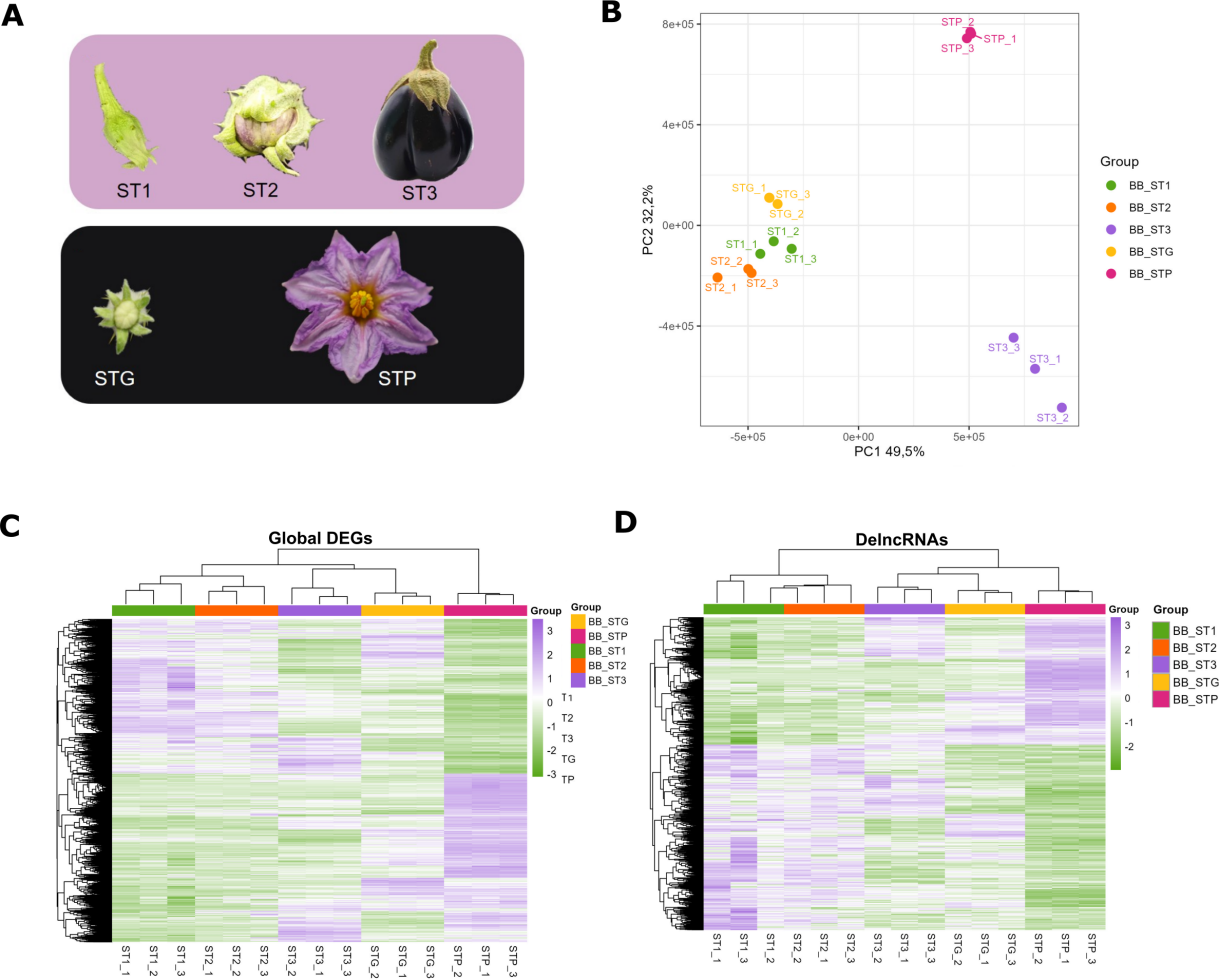
Sample clustering and expression patterns in fruit peel and corolla across developmental stages. **(A)** Developmental stages analyzed in fruits and flowers. Fruit stages are shown in the upper panel: Stage 1, Stage 2, and Stage 3 (ST1, ST2, and ST3). Flower stages are shown in the lower panel: green and purple stages (ST G and ST P). **(B)** Principal component analysis (PCA) of the samples. **(C)** Heatmap of the scaled TPM matrix of all genes across peel and corolla developmental stages. **(D)** Heatmap of the scaled TPM matrix of differentially expressed lncRNAs (DElncRNAs) across peel and corolla developmental stages.

To deeply characterize the anthocyanin biosynthesis and regulation in BB, both berries’ peel and flowers’ corolla tissues were sampled, following the progressive anthocyanin accumulation. RNA-sequencing (RNA-seq) was performed on (i) nine samples of peel (three biological replicates for each of the three stages, ST1 green, ST2 color shifting and ST3 completely purple peels, **Figure 2A**), and (ii) six flower’s corolla samples (three biological replicates of STG, green corolla, and STP, purple corolla, **Figure 2A**), yielding a total of 138 Gb of raw reads. After trimming the adaptors and removing low-quality reads, the number of cleaned reads retained per sample ranged from 46 to 72 million (**Supplementary Table 2**). All 15 samples showed high sequencing quality, with Q30 scores between 91% and 94%, ranging from 6.9 and 10.7 Gb of high-quality reads per sample, which yield a percentage between 90% and 99% of mapped reads on BB genome. After transcriptome assembly, the integrated set of transcripts obtained from all 15 samples included 71, 243 transcripts (**Supplementary Table 2**).

### 3.3 Identification of lncRNAs, new protein-coding genes and protein-coding genes AS events

The transcriptome assembly of the 15 BB RNA samples was obtained to identify lncRNAs, new protein-coding genes and AS events, based on a comparison of the primary BB annotation. The integrated transcriptome assembly set included 71, 243 transcripts, which were then filtered on the basis of their position in the genome.

For lncRNAs, the transcripts were filtered for length > 200 nt, and class codes “i”, “o”, “s”, “u” and “x”, obtaining 5, 604 putative lncRNAs. Of these transcripts, only the ones that presented a coding potential < 0 according to Coding Potential Calculator 2 (CPC2), FlExible Extraction of LncRNAs (FEELnc) and Coding Potential Assessment Tool (CPAT) were selected. After PfamScan filtering on these transcripts, 4, 126 lncRNAs were predicted (**Supplementary Figure 2A**). Of these lncRNAs, 208 belonged to the class code “i”, 946 to the class code “o”, 4, 449 to the class code “u” and only 1 to the class code “x”, while no transcripts were found in class code “s” (**Supplementary Table 3**). Transcripts with coding potential > 0 were also evaluated for protein domains, and a final number of 179 transcripts were considered as novel coding genes (**Supplementary Figure 2B**). For protein-coding genes AS events, the integrated transcriptome was filtered for class codes “n”, “m”, and “j”, amounting to 34, 745 new isoforms detected. After SUPPA analysis, AS events were divided for their AS site type and are listed in **Supplementary Table 3**. The A3 was the most frequent AS type in BB’s peels and corolla, followed by SE, A5 and RI.

The presence of some lncRNAs and protein-coding genes AS events relevant for further analysis has been verified by qPCR (**Supplementary Table 4**).

### 3.4 Identification of differentially expressed genes and lncRNAs

A principal component analysis (PCA), performed on the 15 RNA samples normalized and filtered counts, clearly separated them into the five different groups (ST1, ST2, ST3, STG and STP, **Figure 2B**). The first two principal components (PC1 and PC2) accounted for 81.7% of the variance (**Figure 2B**). For the Differential expression analysis (DEA), coding and novel coding transcripts were filtered for a Log2 fold change (log2FC) of 1, while lncRNAs for a log2FC of 0.5.

Specifically, 855 transcripts were up-regulated and 889 were down-regulated in ST1 compared to ST2 in the peel, whereas 3, 035 transcripts were up-regulated and 2, 854 down-regulated in ST2 compared to ST3 in the same tissue. In the corolla, comparison between STG and STP revealed 5, 685 up-regulated and 5, 227 down-regulated transcripts (**Figure 2C**, **Supplementary Table 5**). Among the DEGs, 28 transcripts in the peel and 51 in the corolla belonged to the novel coding transcripts’ category (**Supplementary Table 5**).

For differentially expressed lncRNAs (DElncRNAs), a total of 683 DElncRNAs were detected in the peel and 1, 120 DElncRNAs in the corolla (**Figure 2D**). Of these lncRNAs, 326 were common DElncRNAs in both peel and corolla (**Supplementary Table 6**). The expression patterns of DElncRNAs were consistent within each sample group, highlighting pronounced differences between peel ST3, corolla’s STG and STP. In contrast, ST1 and ST2 exhibited a similar expression pattern, with only 25 lncRNAs down-regulated and 22 up-regulated between the two ripening stages **Supplementary Table 6**.

**Figure 3:**
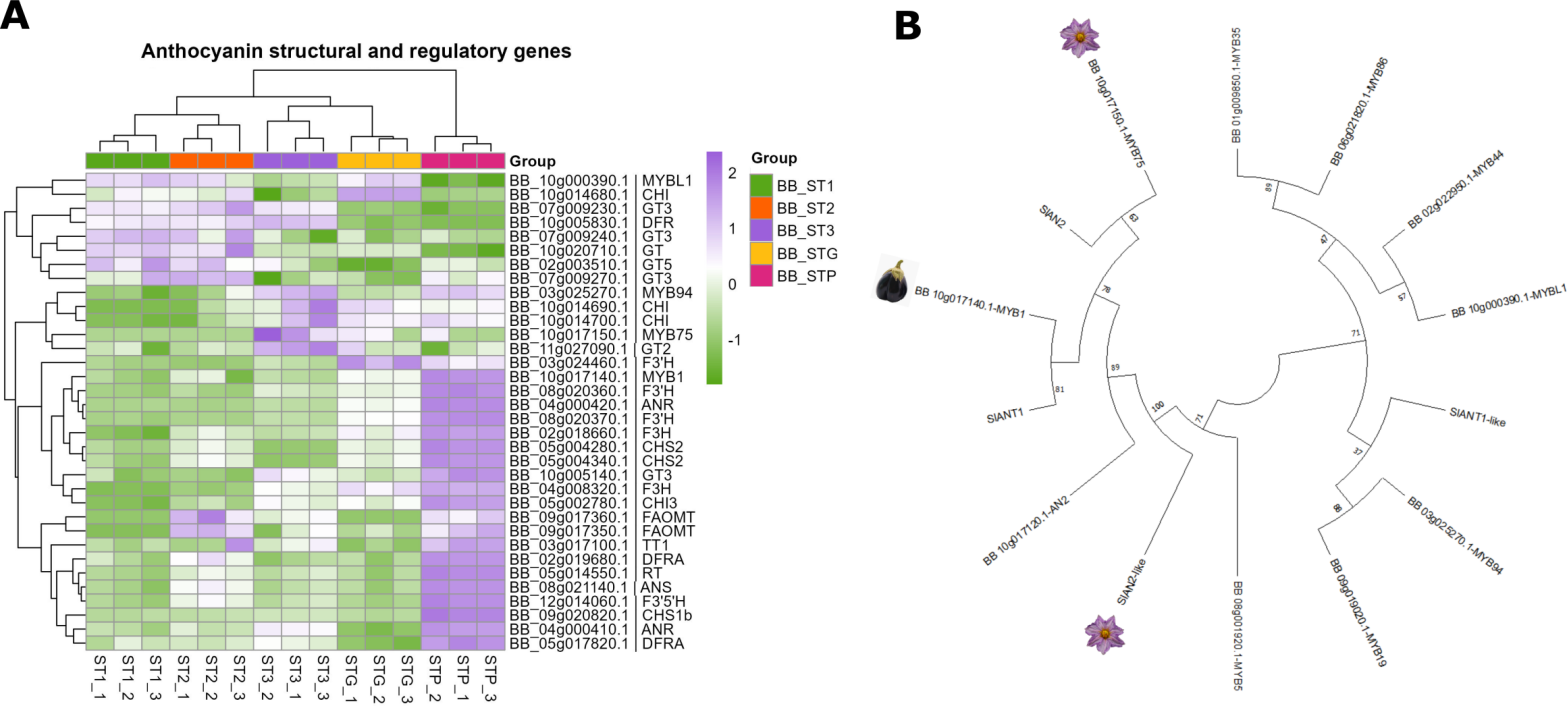
Expression profiling of anthocyanin-related genes and phylogenetic analysis of MYB transcription factors. **(A)** Heatmap of the scaled TPM matrix of structural genes involved in anthocyanin biosynthesis. **(B)** Phylogenetic consensus tree of MYB TFs found to be involved in anthocyanin biosynthesis regulation in eggplant and tomato and realized using neighbor-joining algorithm with 1000 bootstrap. Tissue-specific MYBs are indicated by flower or fruit.

The heatmap in **Figure 3A** illustrates the expression profiles of structural genes involved in anthocyanin biosynthesis. At least one copy of each structural gene was expressed in the purple stages (*i.e.* ST2, ST3, and STP). In STP all structural genes were up-regulated, except *dihydroflavonol 4-reductase* (*DFR;* BB_10g005830.1), which maintained high expression across all peel stages, and some of the *glucosyltransferases* (*GT*; GT2: BB_11g027090.1; GT3: BB_07g009230.1 and BB_07g009240.1).

In the peel, clear stage-dependent differences emerged from the hydroxylation step. In fact, *flavonoid 3′-hydroxylase* (*F3′H*, BB_08g020370.1) was up-regulated in ST3 compared to ST2, whereas, *flavonoid 3′5′-hydroxylase* (*F3′5′H*, BB_12g014060.1) was up-regulated in ST2 compared to ST1. For *chalcone synthase* (*CHS*), BB_05g004280.1 and BB_05g004340.1 followed the same expression trend of *MYB1* in ST2, while *CHS1b* (BB_09g020820.1) was more expressed in ST3. Similarly, among chalcone isomerase (*CHI*) copies, BB_10g014700.1, BB_10g014690.1 and BB_10g014680.1 were more abundant in ST2, while *CHI3* (BB_05g002780.1) increased its expression through the three stages, being up-regulated in ST3. A comparable pattern was observed for the *flavanone 3-hydroxylases* (*F3H*), with BB_02g01860.1 predominantly expressed in ST2 and BB_04g008320.1 in ST3. Likewise, different *dihydroflavonol 4-reductase* (*DFRA*) copies were preferentially expressed in distinct stages, with BB_02g019680.1 dominating in ST2 and BB_05g017820.1 in ST3.

Other structural genes showed limited variation: anthocyanidin synthase (*ANS*) expression remains high and constant from ST1 to ST3, likewise *O-methyltransferases* (*FAOMT*) copies, which catalyze the methylation of delphinidin leading to petunidin and malvidin formation, and cyanidin, leading to peonidin formation (Cammareri et al., 2024), maintained the same trend, suggesting no variation in their expression in the three stages (**Figure 3A**).

Regarding transcription factors, *MYB1* did not show significant regulation in any of the peel purple stages, although a slight, non-significant increase in expression was detectable between ST1 and ST2 (p-value 0.064). By contrast, *MYB1* was significantly up-regulated in the purple stage of the corolla compared to the green one. Similarly, *AN2* and *MYB75* were up-regulated in STP, but showed no expression in the peel, confirming their tissue-specific function. Among minor regulators (Cammareri et al., 2024), *MYB94* was the only activator up-regulated in both ST3 compared to ST2 and STP compared to STG, suggesting its potential involvement in the pathway regulation in both tissues. Conversely, *MYBL1* was down-regulated in ST3 compared to ST2 and in STP compared to STG, while no other minor repressors displayed differential expression (**Figure 3A**).

Because transcription factors involved in anthocyanin biosynthesis may influence pigment accumulation indirectly via hormone-related pathways, genes associated with ethylene and auxin signaling were also examined. In the purple stages of both peel and corolla, particularly in ST3 and STP, several *ERF*s and *Ethylene Insensitive3-Like* (*EIL, BB_06g023930.1*), known enhancers of *ERF* transcription (Feng et al., 2025), were up-regulated compared to green stages, along with *1-Aminocyclopropane-1-Carboxylate Synthase* (*ACS*) and 1*-Aminocyclopropane-1-Carboxylic Acid Oxidase* (*ACO*) transcripts, which are responsible for ethylene biosynthesis. In contrast, transcripts responsible for auxin biosynthesis, *Tryptophan--pyruvate Aminotransferase 1* (TAR1) and *Indole-3-pyruvate Monooxygenase* (*YUCCA*), along with *Auxin Response Factors* (*ARFs*) exhibited higher expression levels in ST1, ST2 and STG, while the *Auxin/Indole-3-Acetic Acid* (*IAA*) transcripts, known as *ARFs* repressors, were predominantly expressed in ST2 and STP (**Supplementary Table 5**).

### 3.5 Phylogenetic analysis of Myb TFs involved in anthocyanin biosynthesis

Since *MYB94* was specifically activated in ST3 and STP, we investigated its phylogenetic placement. Therefore, the main MYB TFs previously reported as activators or repressors in eggplant (Yang et al., 2022; Cammareri et al., 2024) were identified in the BB genome by their protein sequences and annotation. A neighbor-joining phylogenetic tree based on their protein sequences was constructed, including *S. lycopersicum* MYB proteins previously characterized as activators (*SlAN2*, *SlAN2-like*, *SlANT1* and *SlANT1-like*, **Supplementary File S1**). The resulting tree revealed two major clades corresponding to the known functional MYB types. The first clade included the principal activator MYBs in both eggplant and tomato, with MYB1 clustering closely to SlANT1 and MYB75 with SlAN2, as expected. The second clade comprised two subclades: the first one included SlANT1-like, for which no homolog has yet been found in eggplant, clustering with MYB94 and MYB19; the second subclade grouped the repressor MYBL1 with the two minor repressors and MYB35 (**Figure 3B**).

### 3.6 Differentially spliced events in protein-coding genes

**Figure 4:**
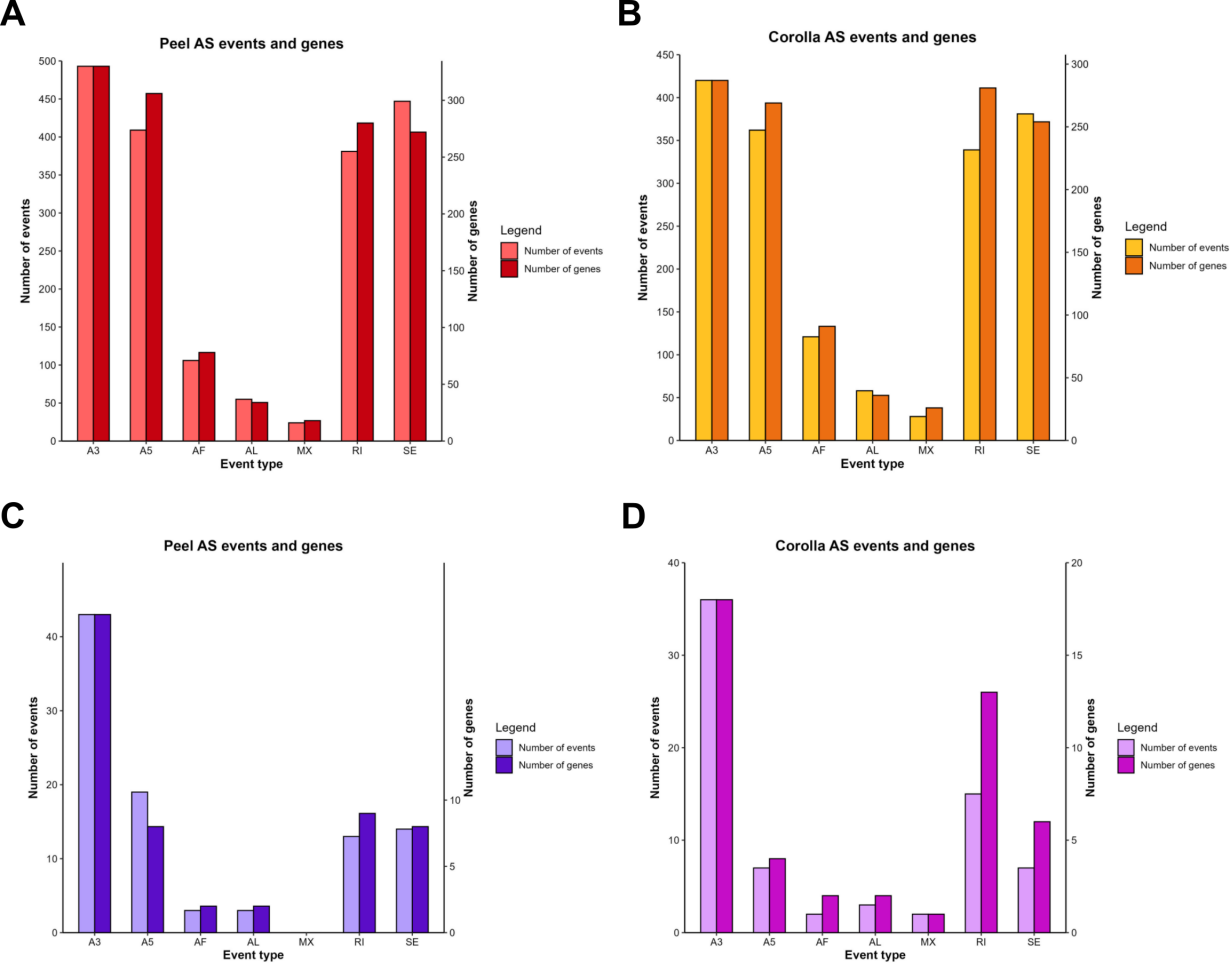
Visual representation DSE of protein-coding genes in BB fruit peel and flower corolla. **(A)** DSE and originating genes detected in the peel. **(B)** DSE and originating genes detected in the corolla. **(C)** DSE and originating genes linked to anthocyanin biosynthesis and regulation detected in the peel. **(D)** DSE and originating genes linked to biosynthesis and regulation detected in the corolla. Alternative 3’ Splice Sites (A3); Alternative 5’ Splice Sites (A5); Alternative First Exons (AF); Alternative Last Exons (AL); Mutually Exclusive Exons (MX); Retained Intron (RI); Skipping Exon (SE).

Concerning AS events, the differentially spliced events (DSE) and their respective alternatively spliced genes were detected: a total number of 1, 915 and 1, 709 DSE were found in the peel and corolla stages, respectively, with 1, 318 and 1, 244 alternative spliced genes. The number of DSE and of differentially spliced genes for each type of event is reported in **Figure 4A**, B for the peel and corolla, respectively.

Among these differentially spliced genes, those linked to anthocyanin biosynthesis or its regulation in peel and in corolla are represented in **Figures 4C, D**, respectively, and listed in **Supplementary Table 7**. Additionally, it was also investigated whether, among these events, differentially spliced genes could overlap with DEGs, particularly focusing on TFs known to be involved in anthocyanin pathway regulation. In the corolla, *MYB75* generated a retained intron (RI) event, retaining both its introns and leading to the loss of the R3 DNA-binding domain and an imperfect version of the R2 domain (**Supplementary Figure 3**). This transcript was up-regulated in STP, although its alternative spliced isoform was more frequent in STG. In the peel, no AS MYBs involved in the anthocyanin regulation were detected, however, an *Ethylene Insensitive3-Like* (*EIL*; *BB_06g023930.1*) gene generating an alternative 5′ splice site (A5) event was identified. The canonical isoform was up-regulated in ST3, whereas the A5-spliced variant was more abundant in ST2. Protein domain analysis revealed no structural differences between the two isoforms.

### 3.7 Network construction and lncRNA interaction with target AS events

To investigate the relationships between DEGs, DSE and DElncRNAs involved in anthocyanin biosynthesis and regulation, a regulatory network was constructed using the Spearman correlation coefficient in order to evaluate potential trans-interaction between transcripts.

**Figure 5:**
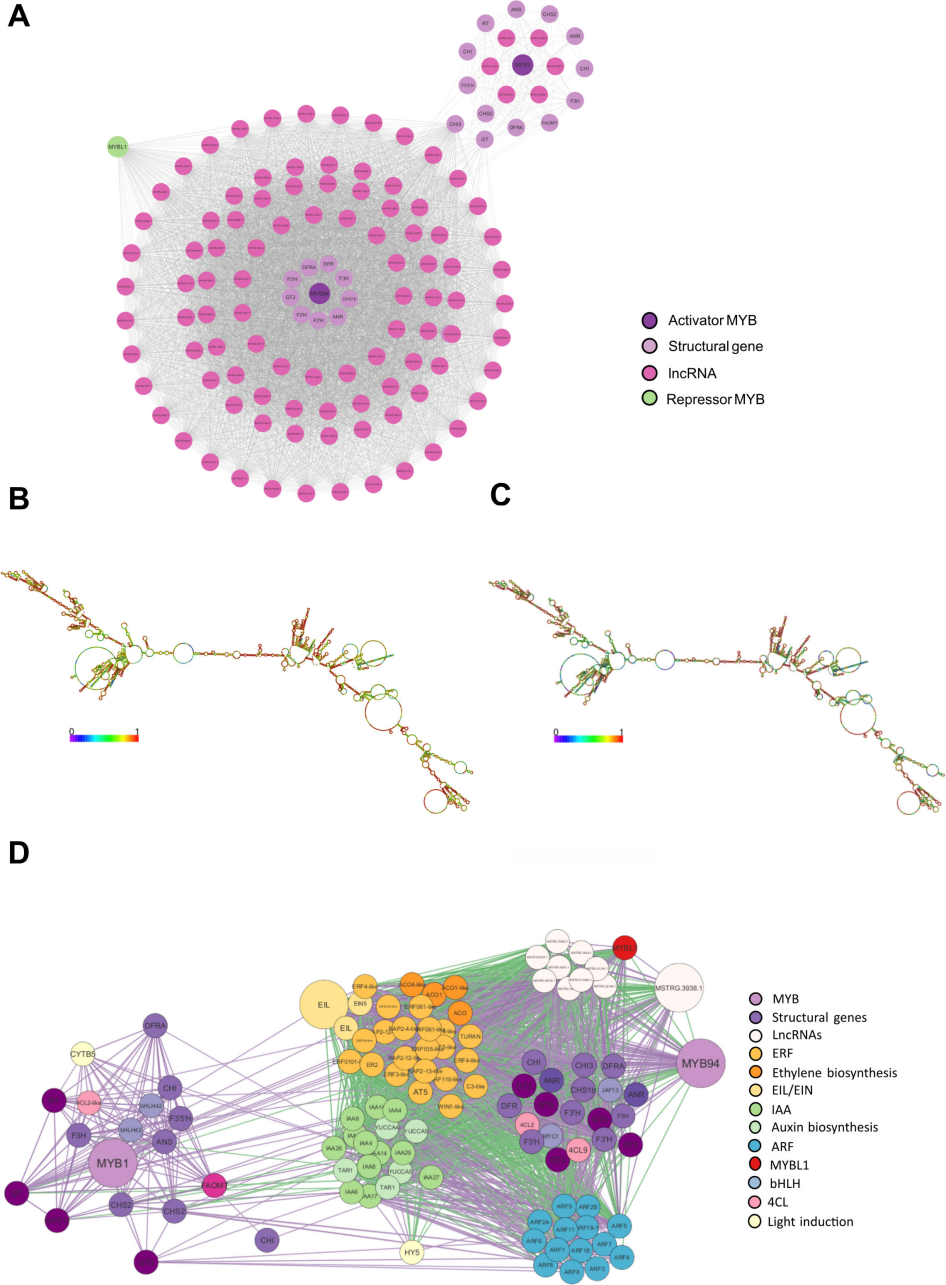
RNA-RNA interaction and interaction networks in BB peel. **(A)** Correlation network representing DElncRNAs interacting with MYB1 and MYB94 and structural biosynthetic genes. **(B)** Centroid secondary structures of MSTRG.3938.1 interacting with EIL (A5). **(C)** Centroid secondary structures of MSTRG.3938.1 interacting with EIL (CDS). **(D)** Correlation network of the DEGs directly or indirectly linked to anthocyanin biosynthesis regulation, interacting DElncRNA and DSE.

First, we selected all the DElncRNAs that displayed a strong correlation with *MYB1*, *MYB94*, *MYB75*, *AN2* and *MYBL1* to understand if they could be involved in the pathway regulation by interacting with these main TFs. In the peel (**Figure 5A**), *MYB94* showed the majority of correlated DElncRNAs, while *MYB1* was in an independent cluster with only six DElncRNAs. Interestingly, the structural genes also clustered differently between the two *MYB*s.

To construct the final network, which involved DElncRNAs and AS events, the ten DElncRNAs with the highest interaction free energy with *EIL* were selected from the 94 initial set (**Supplementary Table 8**). Among them, *MSTRG.3938.1* showed the highest interaction free energy (-88.55 kcal/mol) with both the canonical spliced version and the A5 spliced isoform of *EIL*, determining that the bond took place in a region present in both transcripts (**Figures 5B, C**). MSTRG.3938.1 was negatively correlated with *EIL* and *MYB94*, while exhibiting a positive correlation with *MYBL1*. *MYB94* showed a strong positive correlation with *EIL*, its homologues, several *ERFs* and structural genes of the anthocyanin biosynthesis, including *CHS1b*, *CHI3*, *F3’H* and *DFRA*, all of which were more expressed in ST3 compared to ST2. In contrast, *MYB94* was negatively correlated with *MSTRG.3938.1* and *MYBL1*, which displayed negative correlations with the structural genes as well as with some of the *IAA* and *ARF* TFs down-regulated in ST3 (**Supplementary Table 9**). *MYB1* was only positively correlated to other copies of the structural genes, confirming the presence of a distinct cluster from the *MYB94* one (**Figure 5D**).

**Figure 6:**
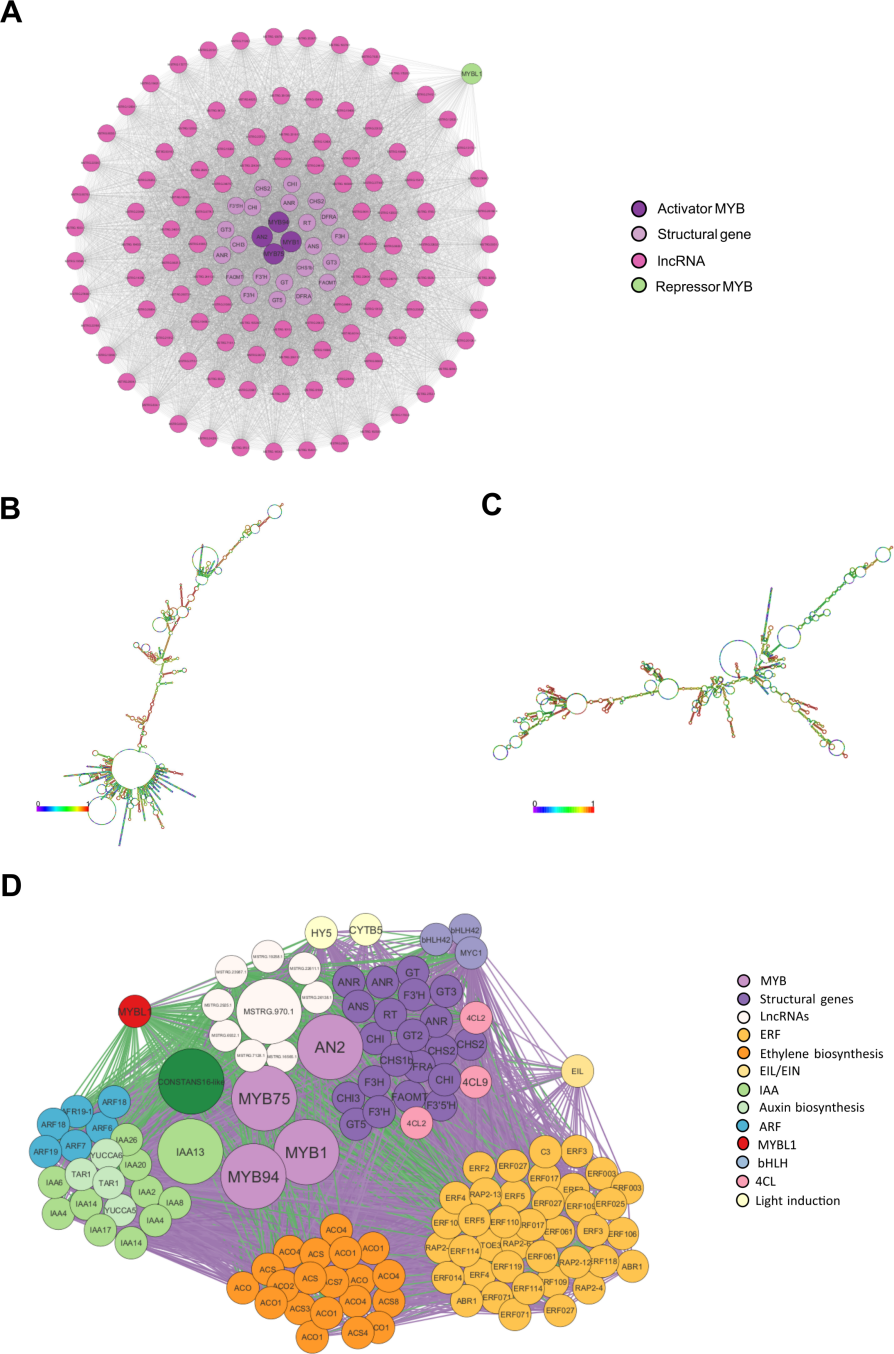
RNA-RNA interaction and interaction networks in BB corolla. **(A)** Correlation network representing DElncRNAs interacting with MYB75, AN2, MYB1, MYB94 and structural biosynthetic genes. **(B)** Centroid secondary structures of MSTRG.970.1 interacting with MYB75 (RI). **(C)** Centroid secondary structures of MSTRG.970.1 interacting with MYB75 (CDS). **(D)** Correlation network of the DEGs directly or indirectly linked to anthocyanins biosynthesis regulation, interacting lncRNA and DSE.

In the corolla, the DElncRNAs correlated with the *MYB* TFs create a more homogeneous network, with several DElncRNAs correlated with more than one TF (**Figure 6A**).

To construct the final corolla network we also selected other TFs, identified as DSE, interacting with *MYB75*. The latter showed a negative correlation with *CONSTANS16-like* (BB_03g028630.1, A3), whose family plays a key role in regulating flowering time and can promote anthocyanin biosynthesis (Liu et al., 2025), and a positive correlation with *IAA13* (BB_09g011750.1, A3). The three TFs were correlated to 39 common DElncRNAs, from which the ten with the highest interaction free energy were selected with the DEGs directly or indirectly involved in anthocyanin biosynthesis regulation (**Supplementary Table 8**). Among them, the lncRNA *MSTRG.970.1* was negatively correlated with *MYB75* and displayed a free energy interaction of -57.22 kcal/mol only with its RI isoform (**Figures 6B, C**). *MSTRG.970.1* also showed the potential to interact with the canonical spliced isoform of MYB75, but with a significantly lower free energy interaction (**Supplementary Table 9**). Furthermore, this lncRNA was negatively correlated with *IAA13*, and positively correlated with *CONSTANS16-like*.

*MYB75* also displayed positive correlation with *AN2*, *MYB94* and *MYB1*, all of which were positively related to different copies of the structural genes. In contrast, *MYB75* was negatively correlated with *MYBL1*, which displayed a negative correlation with *IAA13* and a positive one with *CONSTANS16*-*like* and *MSTRG.970.1.* Moreover, in the corolla, several *ERFs* were positively correlated with *MYB* activators, along with *IAA* TFs (**Figure 6D**).

### 3.8 Isolation, identification and quantification of anthocyanin compounds

**Figure 7:**
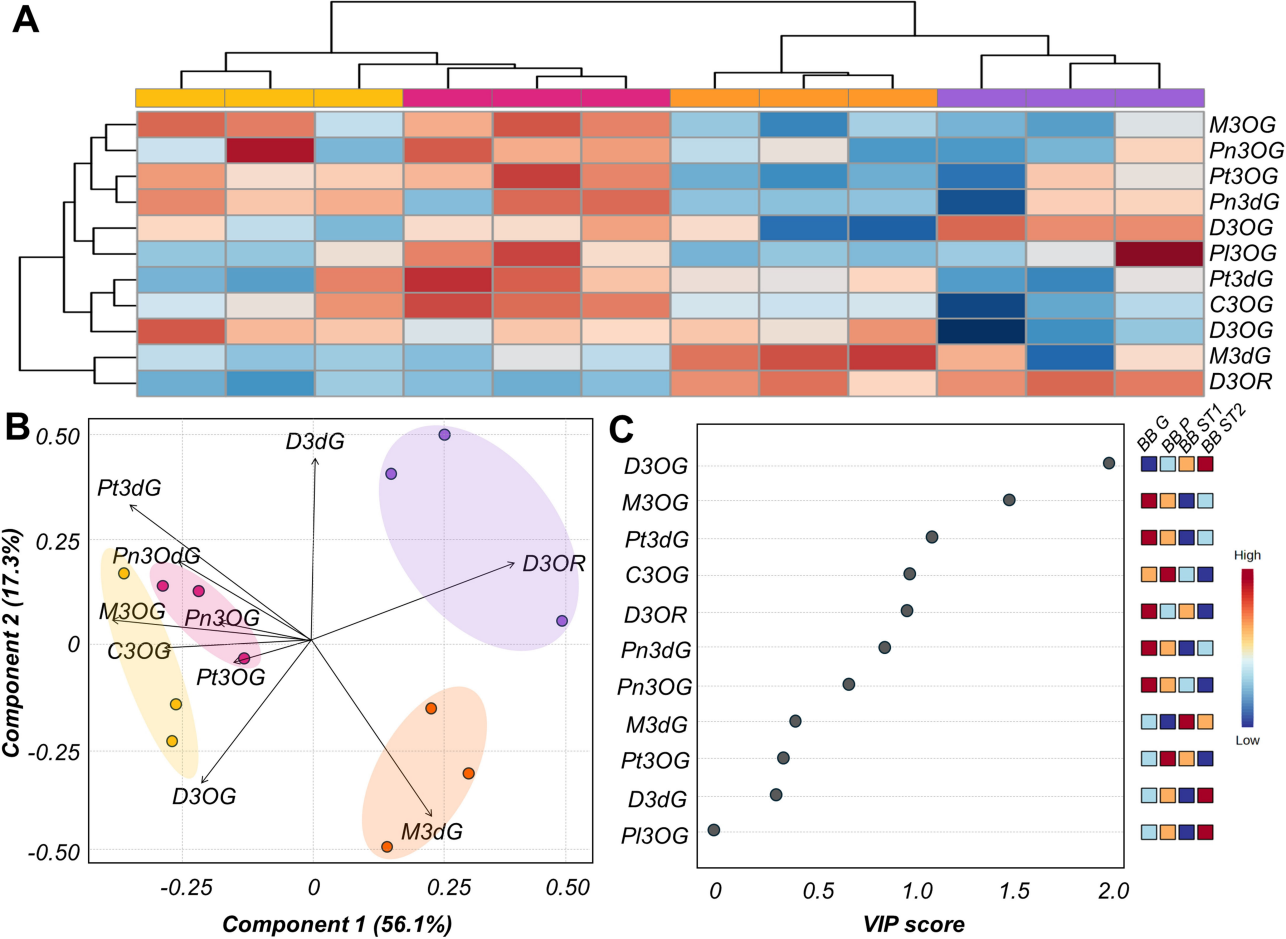
Stage-dependent variation of anthocyanin-related metabolites identified by HPLC-DAD-ESI-MS/MS analysis. **(A)** Heatmap coupled with hierarchical clustering showing the relative accumulation patterns of anthocyanins in BB flower corolla (BB_STG and BB_STP) and fruit peel (BB_ST2 and BB_ST3), highlighting stage- and tissue-dependent differences in metabolite abundance. **(B)** SPL-DA biplot illustrating the separation of samples based on their anthocyanin profiles across developmental stages and tissues. **(C)** VIP score plot identifying the metabolites that contribute most to the observed discrimination among samples.

Overall, anthocyanin-related metabolites displayed clear stage-dependent accumulation patterns, with distinct profiles characterizing corolla and peel samples across development. Specifically, C3OG and Pt3OG are primarily responsible for discriminating between the early stages (STG and STP), while diglucosides and oxidized derivatives, such as D3OR, characterize the more advanced stages (ST1 and ST2). Indeed, in STG samples, Pt3OG and C3OG represent the most significant proportion of total anthocyanins (21.16% and 16.94%, respectively), followed by Pn3OG, M3OG, Pl3OG, and diglucosides, which together contribute less than 15%. In the STP samples, there is a further increase in Pt3OG (27.63%) and C3OG (22.76%), while D3OR shows a slight increase (6.61%), indicating the beginning of the metabolic transition. Concerning the stages ST2 and ST3, the anthocyanin profile undergoes a drastic change. D3OR becomes the predominant compound, increasing from 5–6% in the early stages to over 38–40% in the advanced stages, while C3OG and Pt3OG decrease significantly (C3OG 10.3–8.09%; Pt3OG 18.63–16.11%). Diglucosides show different trends: M3dG increases to 7.22% in ST2 before decreasing slightly in ST3, while D3dG and other diglucosides maintain intermediate values. The forms derived from peonidin and petunidin (Pn3dG and Pt3dG) remain marginal (<3% in ST2 and ST3). As shown in the heatmap coupled with cluster analysis (**Figure 7A**), thanks to different chemical compositions, samples grouped into two different roots. This separation was further confirmed by the biplot originated from SPL-DA analysis (**Figure 7B**), which highlighted the different organization of the samples and further reinforced these observations, allowing for maximum separation between groups based on anthocyanin content. The supervised model showed a clear distinction between the four stages considered. In particular, the division of the samples is clearly delineated along the first two principal components: PC1, which explained 56.1% of the variability, clearly discriminates STG and STP (characterized by negative scores) from ST2 and ST3 (positive scores), while PC2, which explained 17.3% of the variability, further distinguishes ST2 (negative scores) from ST3 (positive scores). Furthermore, VIP (Variable Importance in Projection) values (**Figure 7C**) allowed the metabolites most responsible for the separation to be identified, highlighting how simple glycosides are mainly associated with the early stages, while diglucoside and substituted derivatives characterize the mature stages.

## 4 Discussion

The eggplant anthocyanins biosynthetic pathway has been extensively characterized in terms of both structural genes and their transcription factors (TFs). These TFs belong to the MBW (MYB-bHLH-WD40) complex that has also been deeply studied, leading to the identification of major activators and some repressors within the MYB TF family (Cammareri et al., 2024). However, to our knowledge, only a few studies (Wang et al., 2022a) have explored the alternative splicing (AS) events associated with anthocyanins biosynthesis in eggplant, and no studies have addressed the role of long non-coding RNAs (lncRNAs) in this process.

To expand the understanding of anthocyanin regulation in this species, a *de novo* genome assembly of the “Black Beauty” (BB) cultivar was generated. This high-quality, contiguous assembly provided a robust reference framework for accurate transcript identification and expression profiling within a consistent genetic background. The integrated analysis of BB peel and corolla samples enabled a comprehensive reconstruction of the transcriptome, leading to the identification of 71, 243 transcripts, including 31, 316 genes predicted after the genome annotation, 4, 126 lncRNAs, 179 novel protein-coding genes, and 34, 745 alternative splicing events.

Moreover, the genomic positions of the main structural genes and transcription factors involved in the anthocyanin biosynthetic pathway were found to be conserved between the BB genome and the eggplant reference line “67/3”, except for an inversion of chromosome 10, encompassing three *chalcone isomerase* (*CHI*) gene copies, which did not present any variation in their sequences compared to the reference. Therefore, these structural variations are not associated with anthocyanin production, as both cultivars display purple pigmentation in peel and corolla.

### 4.1 Regulation in fruit peel

The differential expression analysis (DEA) suggested that ST2, corresponding to the early color transition from green to purple in the peel, differed more from ST3 than from ST1. Indeed, only a subset of the anthocyanin biosynthesis structural genes was up-regulated in ST2, correlating with *MYB1* transcription pattern. In fact, *MYB1* expression decreased between ST2 and ST3, as observed for all copies of its correlated structural genes, including *F3′5′H*. These data are consistent with the content of delphinidin-3-O-rutinoside we measured, which was the most present anthocyanin compound in the peel, as expected.

In contrast, *MYB94* expression increases significantly between ST2 and ST3, showing a strong correlation with alternative copies of both EBGs (*CHS1b, CHI*, F3H) and LBGs (*F3′H* and DFRA). Consistently, phylogenetic analysis placed *MYB94* in a subclade together with *SlANT1-like*, a previously characterized activator in tomato (Colanero et al., 2018), suggesting that *MYB94* may represent its eggplant ortholog.

The differential expression of *F3’H* (BB_03g024460.1, BB_08g020360.1, and BB_08g020370.1) *and F3’5’H* (BB_08g020370.1) as well as the different paralogues of *DFR*s (BB_02g019680.1 in ST2 and BB_05g017820.1 in ST3) between ST2 and ST3 could determine a shift in the type of anthocyanin compound produced, as reflected by the slight increase of peonidin-3-O-glucoside and petunidin-3-O-glucoside observed in ST3 samples. These findings are consistent with the recent observations in *Passiflora* spp. where F3′H seems to regulate cyanidin accumulation, F3′5′H determines blue pigmentation, and DFR enhances the biosynthesis of pelargonidins (Nutricati et al., 2025). Moreover, in *Clarkia gracilis*, the spatial and temporal differential expression of the *DFR* paralogues has been reported to influence the anthocyanin-type composition (Martins et al., 2013).

Therefore, we hypothesize that in eggplant fruit peel, two complementary pathways are activated by two distinct *MYB* transcription factors, *MYB1* and *MYB94*, leading to the synthesis of different anthocyanin compounds: delphinidin and cyanidin at two different developmental stages, ST2 and ST3, respectively. This is in accordance with the temporal *MYB*-mediated regulation of anthocyanin synthesis reported in most plants (Schwinn et al., 2006; Albert et al., 2011; Azuma et al., 2015). The possibility that two MYB activators regulate specific paralogous copies of structural genes has already been reported in other species, such as *C. gracilis*, where two MYB activators were found to be expressed at distinct developmental stages, following the same trend as the structural genes activated in each stage (Lin and Rausher, 2021).

Similarly, in *Gerbera hybrida*, the *CHS* gene most closely associated with cyanidin production was *CHS4* (Deng et al., 2014), whose protein sequence shares 88.1% similarity with *CHS1b* in eggplant. This finding supports the idea that *CHS1b*, which is associated with the pathway regulated by *MYB94*, may play a key role in eggplant cyanidin biosynthesis.

**Figure 8:**
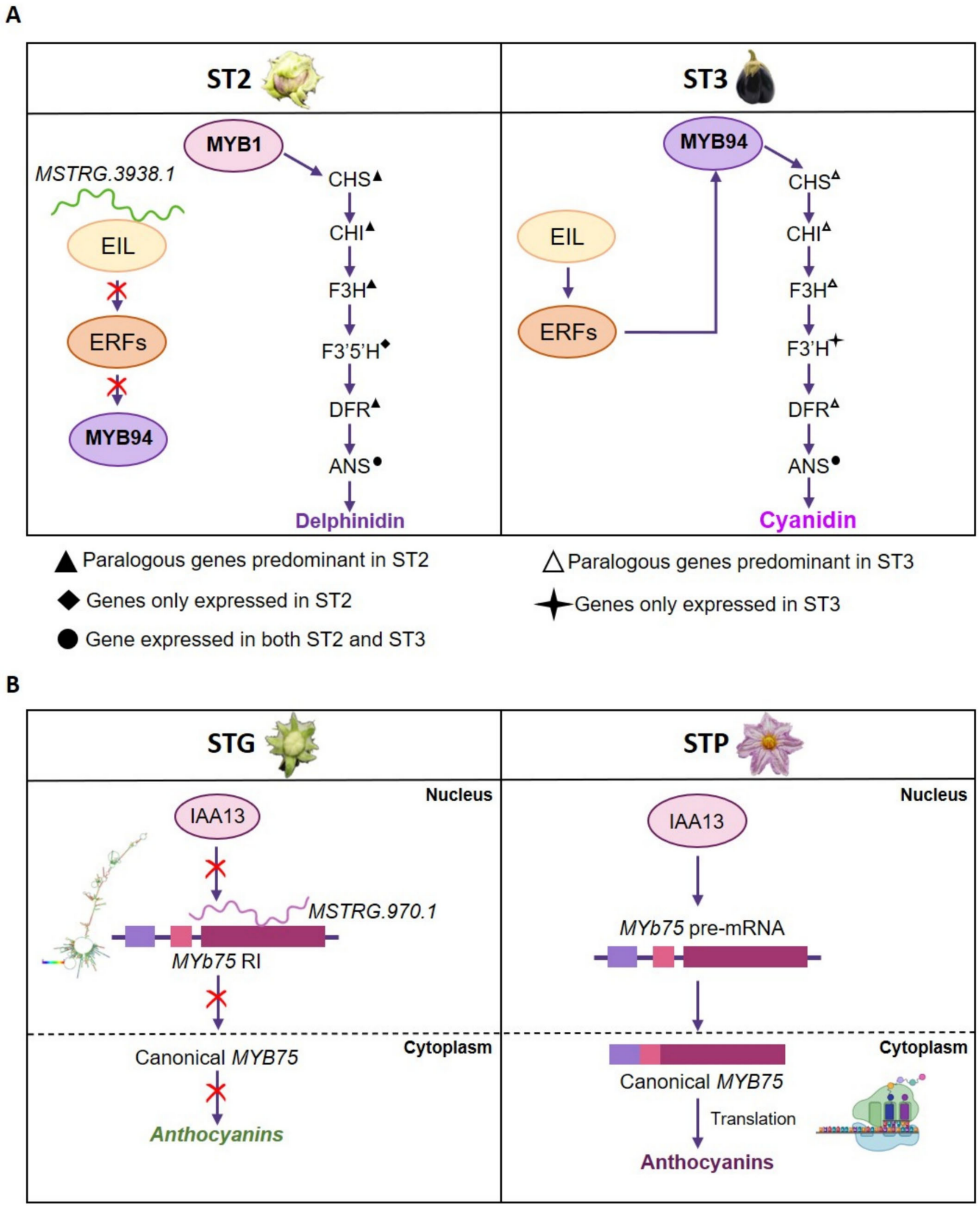
Models of anthocyanins transcriptional regulation in BB fruit peel and flower corolla. **(A)** Peel: in ST2, the lncRNA MSTRG.3938.1 binds EIL inhibiting ethylene signaling cascade and, therefore, MYB94 expression. In this stage, MYB1 and its correlated structural paralogous genes are activated, determining delphinidin production. In ST3, the MSTRG.3938.1 expression decreases significantly, therefore MYB94 is expressed and triggers the activation of other paralogous copies of the structural genes, which implies cyanidin production. **(B)** Corolla: in STG, when the auxin level is higher, the lncRNA MSTRG.970.1 binds to the RI form of MYB75 by sequestering it in the nucleus. The splicing of MYB75 transcript cannot occur, therefore it is degraded or stored until a change in external conditions, preventing anthocyanins biosynthesis. Conversely, in STP, where IAA enzymes are active and the auxin level decreases, the level of MSTRG.970.1 transcript drastically drops, freeing MYB75 transcript and allowing the splicing to occur. The canonical MYB75 determines the production of anthocyanins.

Ethylene has been identified as a positive regulator of the anthocyanin biosynthetic pathway in several species, such as apple and strawberry, both producing cyanidin (Wang et al., 2022b), suggesting a specific involvement of this hormone in its synthesis. Although eggplant is a non-climacteric fruit, ethylene is marginally produced during the berries’ maturation, and can contribute to the ripening process (Fan et al., 2016). In our study, transcriptomic evidence indicated a potential increase in ethylene biosynthesis at ST3, as suggested by the up-regulation of several ethylene biosynthesis structural genes (*e.g.* 1-Aminocyclopropane-1-Carboxylate Synthase, *ACS*, and 1-Aminocyclopropane-1-Carboxylic Acid Oxidase, *ACO*) and *Ethylene Response Factor* (ERF) transcription factors (TFs). These transcripts were positively correlated with *Ethylene Insensitive3-Like* (*EIL*) and *MYB94*, suggesting a role for ethylene in activating the alternative pathway proposed (**Figure 8A**). Our analysis revealed that, in the peel, *EIL* underwent an Alternative 5’ Splice Site (A5) event. The A5 isoform conserved all the protein domains of its canonical version and was predominant in ST2, where *EIL* expression is down-regulated compared to ST3. *EIL* genes are known as activators of *ERF* TFs, which can also promote the ripening stage in several plant species, including tomato (Gu et al., 2017). Recently, it has been proposed that ERF proteins can interact with MYB TFs in eggplant, forming complexes aiming to activate anthocyanin structural genes in response to light signaling (Li et al., 2025). In ST3 we have not observed an increase in *HY5* transcript, which was instead strongly expressed in ST2, therefore, we cannot exclude a light–dependent mechanism regulating the increase of ethylene biosynthesis detected in ST3.

In apple, it has been reported that lncRNA could influence the anthocyanin production, in particular the expression of the *lncRNA MdLNC610* regulat*es* the transcription of the ethylene biosynthetic gene *MdACO1*, resulting in the accumulation of cyanidin in fruit peel. In our study we identified a lncRNA (*MSTRG.3938.1)* upregulated in ST2 that is predicted to bind both the A5 and the canonical EIL isoforms, potentially influencing its transcription and consequently the ethylene-signaling cascade. Moreover, *MSTRG.3938.1* was found negatively correlated also with *MYB94*, but positively with the R3-MYB repressor *MYBL1* which is down-regulated in ST3. These findings suggest that the lncRNA-mediated modulation of *EIL* and the *MYBL1* activity may indirectly influence *MYB94* expression and, consequently, the transcription of its correlated structural gene.

### 4.2 Regulation in flower

In corolla, the DEA highlighted the expression of *AN2* and *MYB75* in STP, which were absent in the peel, confirming their tissue-specific activity (Cammareri et al., 2024). Also, *MYB1* and *MYB94* were expressed in the STP, but in lower level compared to the other two *MYBs*. Among the structural genes and MYBs, their differences in the expression are not correlated with the composition of the final anthocyanin compounds produced, since all the paralogues of the structural genes were positively correlated with at least one of the *MYB*s. The metabolic analysis confirmed that, also in the corolla, the total anthocyanin content increased in STP compared to STG, identifying cyanidin-3-O-glucoside and petunidin-3-O-glucoside as the most relevant compounds.

In plants, pigmentation has been demonstrated to be regulated by AS of the TF responsible for the activation of colored molecule biosynthetic genes. For instance, in tomato, *SlAN2like* gene displayed a splicing mutation responsible for the loss of function of its protein in wild-type plants, leading to the lack of anthocyanin pigmentation in cultivated genotypes (Colanero et al., 2020).

Similarly, in grapevine, the exon skipping splicing event of *VvMYBA1-L* resulted in decreased accumulation of anthocyanin in grape berry flesh (Gao et al., 2023).

Interestingly, in our corolla samples we observed a differential AS event in *MYB75*, specifically a retained-intron (RI) isoform that was enriched in STP.

In plants, RI represents a major regulatory mechanism that influences TF expression by controlling the fate of their pre-mRNA. In fact, this regulation can occur through nuclear retention of RI transcripts that prevent TF translation (Boothby et al., 2013). This mechanism has been demonstrated to be controlled by the action of lncRNAs that can hybridize with the pre-mRNAs to modulate their splicing dynamics (Pisignano and Ladomery, 2021).

Notably, *MYB75* showed a negative correlation with the lncRNA *MSTRG.970.*1, whose RI isoform displayed a predicted strong interaction with, suggesting a regulatory relationship in which *MSTRG.970.1* binds the RI isoform, hindering its maturation in STG. This interaction could lead to the degradation of IR *MYB75* transcripts via nonsense-mediated decay, or could be responsible for the storage in the nucleus of the IR *MYB75* isoform. This transcript could be converted into mature spliced *MYB75* at a specific time during development, enabling translation to proceed. In fact, in STP, the lncRNA *MSTRG.970.1* is downregulated and this could allow the proper *MYB75* splicing, and, consequently, the activation of anthocyanin biosynthesis in flowers.

Previous studies in various systems support this model (Jia et al., 2020). Zhou and collaborators demonstrated that in *Arabidopsis*, *COP1* is involved in light-regulated IR to modulate seedling photomorphogenesis. IR leads to the nuclear detainment of IR transcript as storage and, and upon changes in environmental stimuli or developmental phases, they can be fully spliced and translocated into the cytoplasm for translation (Zhou et al., 2024). Moreover, in *Marsilea vestita*, a large number of intron-containing, polyadenylated pre-mRNAs have been demonstrated to be stored in the microspore and their maturation during development enabled their translation (Boothby et al., 2013).

Moreover, in apple, the regulatory effects of auxin signaling on anthocyanin metabolism have been investigated showing that exogenous auxins repress anthocyanin biosynthesis, most likely through the MdIAA121–MdARF13 signal transduction pathway (Wang et al., 2018). In our analyses, we found that *IAA13* is positively correlated with *MYB75*, and both share correlations with the same lncRNAs, suggesting a common regulatory pathway.

Taken together, these data are consistent with a model in which *MSTRG.970.1* modulates *MYB75* post-transcriptionally and possibly influences a broader hormone-linked regulatory module by the interactions with auxin repressors. This mechanism likely contributes to the switch from a *MYB75*-associated state in green stages to an active anthocyanin-production state in STP (**Figure 8B**).

## 5 Conclusion

This work confirms the tissue-specific roles of eggplant key TFs and sheds light on the involvement of the minor activator *MYB94* in regulating anthocyanin biosynthesis, supporting transcriptional data with complementary metabolite analyses. LncRNAs and AS events directly correlated with anthocyanin accumulation were identified, enabling the delineation of tissue-specific interaction models for fruit peel and flower corolla with the involvement of hormone signaling components. Altogether, these results provide a coherent systems-level view of how transcriptional and metabolic layers converge to control anthocyanin biosynthesis and regulation in BB, starting to uncover the fine-tuned regulatory mechanisms underlying this pathway. Future work will further dissect the functional interactions between the identified lncRNAs and their target TFs and experimentally confirm the regulatory roles suggested by our transcriptomic analyses.

## Data Availability

Raw sequencing data have been deposited in the NCBI Sequence Read Archive (SRA) under BioProject accession number PRJNA1374051. Genomic data, including genome assembly and annotation, have been deposited in the Zenodo repository (https://zenodo.org/records/17790745).
