# Supplementary material for: Transcriptomic landscapes of tissue-specific color transition in eggplant reveal regulatory roles of lncRNAs and alternative splicing in anthocyanin biosynthesis
Source: Front Plant Sci. 2026 May 28;17:1832029. doi: 10.3389/fpls.2026.1832029 (PMC13253822; doi:10.3389/fpls.2026.1832029)
Supplement: Supplementary file 1 [file DataSheet1.docx]

>BB_01g009850.1-MYB35

MGRHSCCYKQKLRKGLWSPEEDEKLIKHITKFGHGCWSSVPKLAGLQRCGKSCRLRWINY

LRPDLKRGTFSQDEENLIIELHAVLGNKWSQIAARLPGRTDNEIKNLWNSSLKKKLRQRG

IDPNTHKPLSEVESEDKASANSNNKNNDKVSESSNNEFNFVDAHENGFSTDKSIIKPAAS

SMINNMERYPLIHEANNIAPPTHEFFTTNCKSPDLSSYLSFHNYNPNTNILFNTKTSTSA

DNMVSDHQFNCSSLPNATFSSMSSILSTTISPLARTFNINKFQNWEACTISSNGSNNSNG

TSNSIELQSNCSFFDNNAAATAFAWGPAGATAADGSGKSEREEIKWSEYLQTPFSLGDNN

NTIDNHHQIPPHQELYDGQTKSETQFMTQGSWLQNQTPQTSLQTTDLYGNNNFQRLPAVF

GQFF

>BB_02g022950.1-MYB44

MASTCNSSKRDMDRVKGPWSPEEDELLQQLVQKHGPRNWSLISKSIPGRSGKSCRLRWCN

QLSPQVEHRAFTPEEDETIIRAHARFGNKWATIARLLNGRTDNAIKNHWNSTLKRKCSSL

SADEGNELADQIFENQQPPLKRSVSAGSAMPVSGFHFSPGSPSGSDSDSSLHVTSSSQSH

VFKPVARTGGIFPPSIDISSPLVDPPTSLSLSLPGVDSAECSNRSSESTQSKNPFQLMLP

AMQIPPPPPPPPPPSQSTAVPFERVSAIQQSHQNPAGVSEQPDKVFVPFSKELLGVMQEM

IKTEVRNYMIGIEQRHQQQQQQQHYQQQQQFQQPSHKQPSGLGLGLCMQQASDGFRDRAA

NRMGISKFD

>BB_03g025270.1-MYB94

MGRPPCCDKIGVKKGPWTPEEDIILVSYIQQHGPCNWRAVPSNTGLLRCSKSCRLRWTNY

LRPGIKRGNFTEHEEKMIIHLQALLGNRWAAIASYLPQRTDNDIKNYWNTHLRKKLKKLQ

GIDENNTQEGTASSSSQSNFSKGQWERRLQTDIHTARKALCEALSLDKSDSPGNPISPIA

AQQPVSGSSSYASSAENISRLLQNWMKNSPKSSELSRSNSETTQSSLNNPSSIGSGSGLS

SSPSEGTISAATPDGFDTFFSEGSAFTPENSAIFQVESKPNFPNMNSENGFLLQSESKTS

LEESQVPLTLLEKWLFDDAINAPGQEEFMAIGLGESADFF

>BB_06g021820.1-MYB86

MGRNSVVVKEKTRKGLWSPEEDEKLYNYITRFGVGCWSSVPKLAGLQRCGKSCRLRWINY

LRPDLKRGMFSQEEEDMIIAVHELLGNRLWGILAAQLVDYLNSHLLGEWAQIAAKLPGRT

DNEIKNYWNSYLKKKLIKQGIDPNTHKPLSENHQVRNETNCTDKASSLLMPKLPNMSNSS

ESEQPFHFNSKRIFNSEAITRQLTEVSRNQLVSKQVFDPLFLYEFQANMNQIGPYGDHHN

QIEGNQDFGFCSMPSLTNFQHGHMTTESDFSDSSTSRMSTSNSSNTMISHYSSAGNQMSE

MLEWNDNKIDSLFQYPYVGIKNEENFSNNNP

>BB_08g001920.1-MYB5

MGRAPCCAKEGLRKGPWSTKEDLLLTNYIKENGEGQWRNLPNKAGLLRCGKSCRLRWMNY

LRPGIKRGNFSQDEEDLIIRLHSLLGNRWSLIAGRLPGRTDNEIKNYWNTHVIKKLKIAG

IQPKVAKKEPKKKPKIEKPTDKQKKNTKKKDQCLVQKTSDHTPQVVFVPKPIRISSGLLR

SSSVEDVALLGTSSLCCSDSPKKLLHPCQSLQKSTTLEESDEQLSIFLKSSSNIASPSDS

SEELAKNNKDINIITKDDENIEGKIEEISFIPRALNLLFDEVLLDGFCDLSNESMLEKVY

EEYLQLISEKCYHLDDPMLL

>BB_09g019020.1-MYB19

MVRAPCCEKMGLKKGPWTPEEDQILISYIQSNGHGNWRALPKLAGLLRCGKSCRLRWTNY

LRPDIKRGNFTREEEDSIIQLHEMLGNRWSAIAARLPGRTDNEIKNVWHTHLKKRLKNYE

PPQNSKRHSKNKNDSKAPSTSQIALKSSHNFSNIQEDINGLRPESGPNSPQLSSSEMSTV

TANSLAATMGVTNSSDSSENYIPEIDESFWTDDLSTTDNSNFDMMVGGGDDLQFHDMKQE

SVEMDVGAKLEDDMDFWYNVFIKSGDLFELPEF

>BB_10g000390.1-MYBL1

MSKDKIQEDWCKDDGMLGNFSEEEGDLIIRFYALLGERWSLIAERLPGHSDDEVKNYWNS

YLKLKLIKMGIDPMNYHIQEYVHKKNLEYFSSRNKKFNADASDAESSSAH

>SlANT1-like

MSRKPCCVGEGLKKGAWTTEEDKKLISYIHDHGEGGWRDIPQKAGLKRCGKSCRLRWTNY

LKPEIKRGEFSSEEEQIIIMLHASRGNKWSVIARHLPRRTDNEIKNYWNTHLKKRLMEQG

IDPVTHKPLASSSNPTVDENLNSPNASSSDKQYSRSSSMPFLSRPPPSSCNMVSKVSELS

SNDGTPIQGSSLSCKKRFKKSSSTSRLLNKVAAKATSIKDILSASMEGSLSATTISHASF

FNGFTEQIRNEEDSSNTSLTNTLAEFDPFSPSSLYPEHEINATSDLNMDQDYDFSQFFEK

FGGDNHNEENSMNDLLMSDVSQEVSSTSVDDQDNMVGNFEGWSNYLLDHTNFMYDTDSDS

LEKHFI

>SlAN2-like

MEGSSKGLRKGAWTAEEDSLLRQCIGKYGEGKWHQVPLRAGLNRCRKSCRLRWLNYLKPS

IKRGKFSSDEVDLLLRLHKLLGNRWSLIAGRLPGRTANDVKNYWNTHLSKKHEPCCKTKI

KRINIITPPNTPAQKVDIF

>SlANT1

MNSTSMSSLGVRKGSWTDEEDFLLRKCIDKYGEGKWHLVPIRAGLNRCRKSCRLRWLNYL

RPHIKRGDFEQDEVDLILRLHKLLGNRWSLIAGRLPGRTANDVKNYWNTNLLRKLNTTKI

VPREKINNKCGEISTKIEIIKPQRRKYFSSTMKNVTNNNVILDEEEHCKEIISEKQTPDA

SMDNVDPWWINLLENCNDDIEEDEEVVINYEKTLTSLLHEEISPPLNIGEGNSMQQGQIS

HENWGEFSLNLPPMQQGVQNDDFSAEIDLWNLLD

>SlAN2

NCIQKYGEGKWHLVPARAGLNRCRKSCRLRWLNYLRPHIKRGDFAPDEVDLILRLHKLLG

NRWSLIAGRXPGRTANDVKNYWNTHFHKKLSIIAPHLHPHPRPRSHPRLQIKHKSIAVTK

NEIIRPQPRNFSNVKKNDSHWCNNKSMITNTSDKDDKRCNEIVVNICEKPIGENTSSIDD

GVEWWTNLLENCIEIEEETANTNFGKTPTMLLHEEISPPLINDEDNSMQQGPTNNWDDFS

TDIDLWNLLN

>BB_10g017140.1-MYB1

MNNPPIICTSVRVRKGSWTEEEDLLLRKCMEKYGEGKWHLVPARAGLNRCRKSCRLRWLN

YLRPHIKRGDFASDEVDLILRLHKLLGNRWSLIAGRLPGRTANDVKNYWNTNLLRKFTIA

PQKINNTCKDIISTNEIIRPQPRKYLSSIKKNNLTNNNVIVDKEERCKEITSDKQTTDAS

MDNGDQWWKSLLENFNDDAVEGEEEAVTNYEKTLTSLLHEEISSPPLNGGGNSMQQEQCD

NWDDFSADIDLWNLLD

>BB_10g017120.1-AN2

MNTATVAKSLGVRKGAWTEEEDLLLRKCMDKYGEGKWHLVPTRSGLNRCRKSCRLRWLNY

LRPHIKRGDFAPDEIDLILRLHKLLGNRWSLIAGRFPERTANDVKNYWNTHIQKKLTNSR

PQMQERKHNNALKITKNTILRPQPRPPPPPPPPPPPPPRTFSSAKNVSWCTNKNMNITNT

LDKDNERHKEIGVNTCEKPKGDATSSSIDDDGVQWWTSLLENCNEIEEEATAVLSFEEEN

KFLPNLLHEENNSPPMQQGQNDGWDDFSVDIDLWNLFN

>BB_10g017150.1-MYB75

MNNPPIMCTSLGVRKGSWTEEEDCLLRKCIQKYGEGKWHLVPARAGLNRCRKSCRLRWLN

YLRPHIKRGDFAADEIDLIMRLHKLLGNRWSLIAGRLPGRTANDVKNYWNTHFQKKLNTI

TASFPSPRKKINNKCIAISKNEIIRPQPRNFSSIKKNISYWCNNNKSMITNTLYKDGNRC

KDIEVNICDKPTGEITSSFEGDGVQWWTNLLENCNEFEHEVDVTNYEKTSTRLLHEEISP

PLGNGEASNFMQRRQSDDWDDFSFDVDLWNLLD
