# Supplementary material for: Transcriptomic landscapes of tissue-specific color transition in eggplant reveal regulatory roles of lncRNAs and alternative splicing in anthocyanin biosynthesis
Source: Front Plant Sci. 2026 May 28;17:1832029. doi: 10.3389/fpls.2026.1832029 (PMC13253822; doi:10.3389/fpls.2026.1832029)
Supplement: Supplementary file 2 [file Image1.pdf]

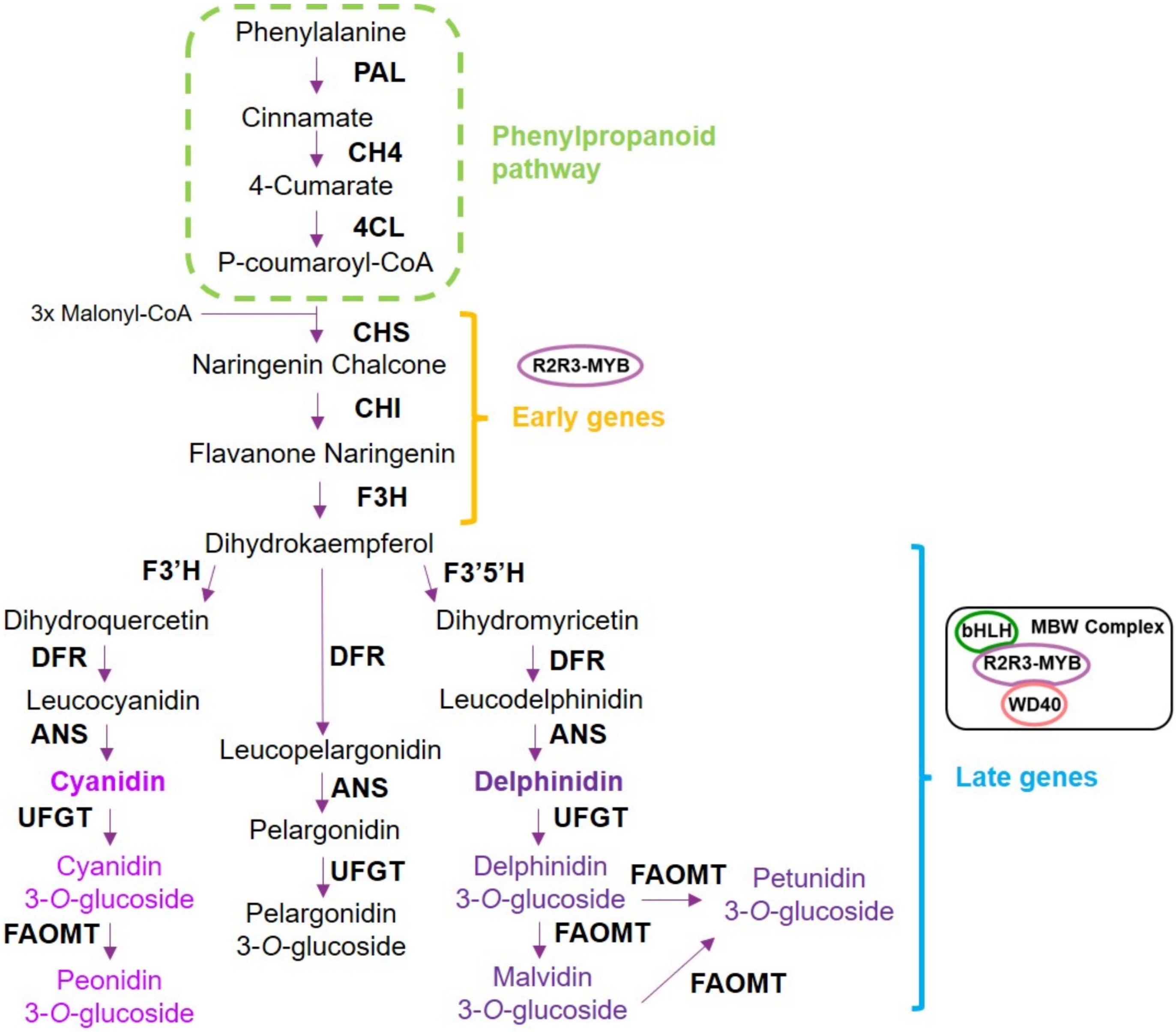


**Figure S1. Anthocyanins biosynthetic pathway.** The diagram illustrates the main intermediates and enzymes involved in the synthesis of anthocyanins from phenylpropanoid precursors, highlighting the key stages during early and late stages in the production of flavonoid pigments in plants.
