# Supplementary material for: Transcriptomic landscapes of tissue-specific color transition in eggplant reveal regulatory roles of lncRNAs and alternative splicing in anthocyanin biosynthesis
Source: Front Plant Sci. 2026 May 28;17:1832029. doi: 10.3389/fpls.2026.1832029 (PMC13253822; doi:10.3389/fpls.2026.1832029)
Supplement: Supplementary file 3 [file Image2.pdf]

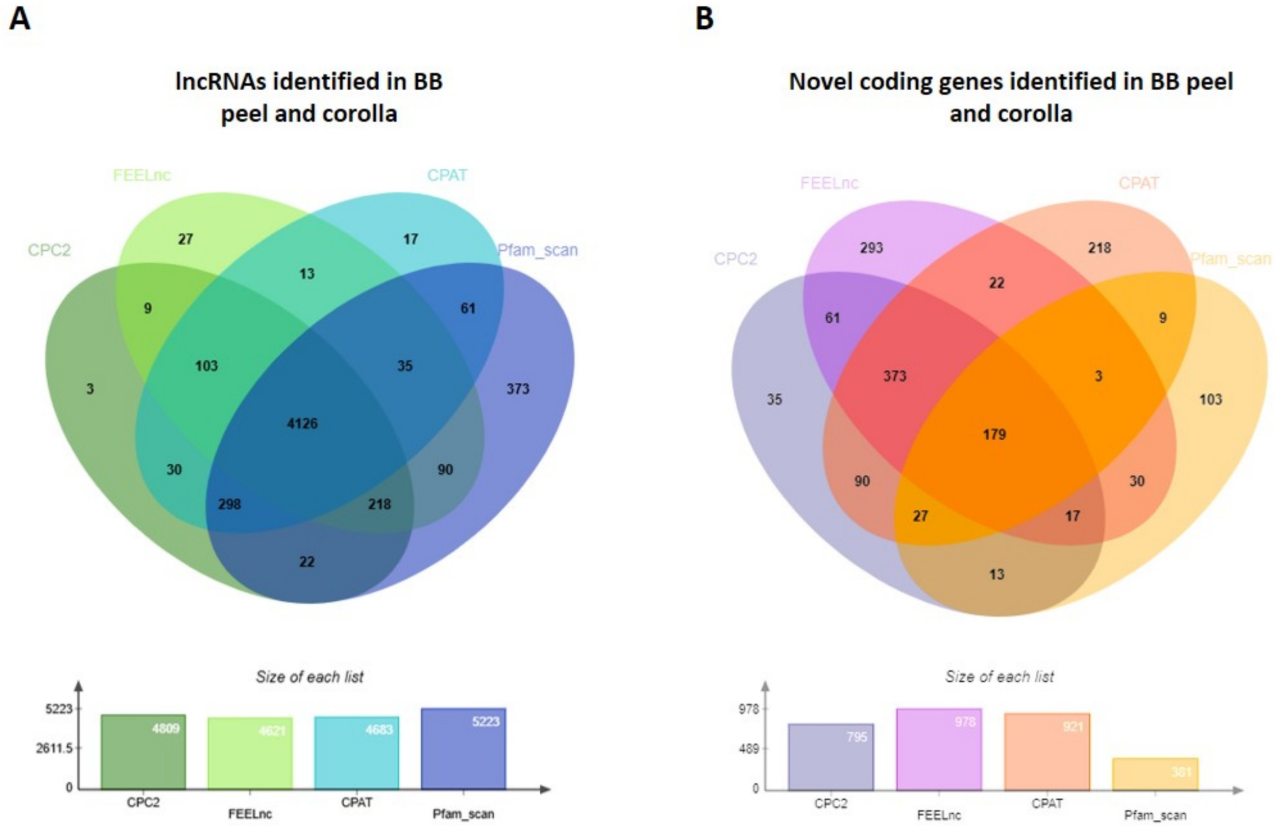

Figure S2. Coding potential assessment with CPC2, FEELnc and CPAT supported by Pfam on the complete set of BB peel and corolla transcripts A *The Venn diagram represents the intersection of all lncRNAs identified by the four programs by coding potential < 0 and the presence of protein domains. The count of the lncRNAs identified by each tool is represented in the bar plot on the bottom* ***B*** *The Venn diagram represents the intersections of novel coding genes predicted by coding potential > 0 and the presence of protein domains. The bar plot at the bottom shows the number of new coding genes identified by each tool. The Venn diagram design was performed with E Venn (http:// www. ehbio. com/ test/ venn/#/).*
