# Supplementary material for: Transcriptomic landscapes of tissue-specific color transition in eggplant reveal regulatory roles of lncRNAs and alternative splicing in anthocyanin biosynthesis
Source: Front Plant Sci. 2026 May 28;17:1832029. doi: 10.3389/fpls.2026.1832029 (PMC13253822; doi:10.3389/fpls.2026.1832029)
Supplement: Supplementary file 4 [file Image3.pdf]

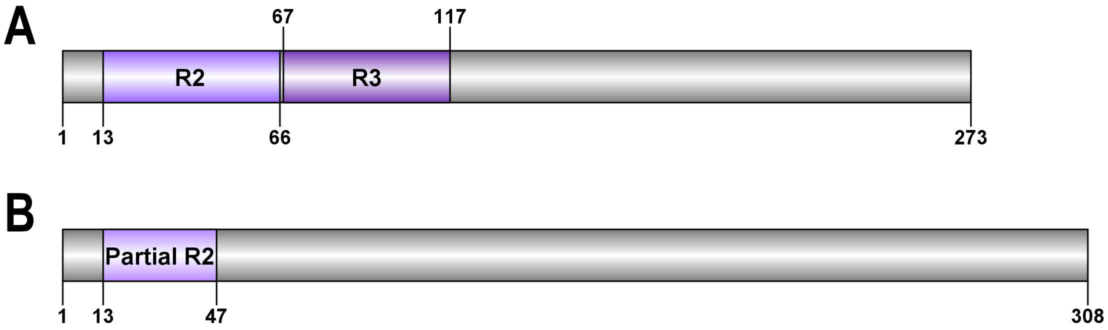

Figure S3: Predicted domain organization of the two* MYB75 *isoforms. A *Schematic representation of the R2R3 MYB domains in the spliced* MYB75 *isoform.* ***B*** *Schematic representation of the incomplete R2 domain in the retained-intron (RI)* MYB75 *isoform. Domain prediction was obtained using InterProScan (https://www.ebi.ac.uk/interpro/result/InterProScan/).*
